# Multi‐Energy Integrated Photocatalysis for Antitumor Therapy

**DOI:** 10.1002/EXP.20240188

**Published:** 2025-10-25

**Authors:** Li Zhang, Jun Liu, Yuxuan Xiong, Guangfu Liao, Yonggang Lv

**Affiliations:** ^1^ State Key Laboratory of New Textile Materials and Advanced Processing Wuhan Textile University Wuhan P. R. China; ^2^ Department of Chemistry The University of Chicago Chicago Illinois USA; ^3^ College of Material Engineering Fujian Agriculture and Forestry University Fuzhou China

**Keywords:** cancer nanotheranostics, piezo‐photocatalysts, photothermal‐photocatalysts, reactive oxygen

## Abstract

Photocatalysis has been recognized as a promising approach in cancer theranostics over the past years. To enhance therapeutic outcomes and overcome current limitations, significant attention has been directed toward the development of multi‐energy integrated photocatalytic systems, particularly piezo‐photocatalysts and photothermal‐photocatalysts. Piezo‐photocatalysis combines the piezoelectric effect with photocatalysis, offering superior efficiency, targeted action, and improved safety compared to traditional photocatalytic methods. By harnessing both mechanical and optical stimuli, this approach enables more precise and effective cancer therapies. On the other hand, photothermal‐photocatalysis integrates heat induced by light with photocatalytic reactions, accelerating reaction rates and promoting the generation of reactive oxygen species. The synergistic interaction of heat and photocatalysis enhances tumor cell apoptosis more effectively than either modality alone. This review provides a systemic overview of the emerging multi‐energy integrated photocatalytic strategies for cancer treatment. It begins with an exploration of the principles of piezo‐photocatalysis and its potential to improve cancer therapies such as piezoelectric‐enhanced single‐modal photodynamic therapy (PDT), dual‐modal sono‐photodynamic therapy, and triple‐modal hydrodynamic therapy/gas therapy (GT)/chemotherapy. Next, we delve into photothermal‐photocatalysis and examine how its integration with additional treatment modalities, such as dual‐modal photothermal/photocatalytic therapy (PTT/PCT) and PTT/PDT, can enhance therapeutic efficacy. Furthermore, we discuss more complex multi‐modal treatments, including triple‐modal PTT/PCT/chemotherapy, PTT/PCT/chemodynamic therapy (CDT), PTT/PCT/GT, PTT/PCT/immunotherapy (IT) and tetra‐modal PTT/PCT/CDT/chemotherapy, PTT/PCT/CDT/ferroptosis therapy (FT), PTT/PCT/FT/IT, and PTT/PCT/GT/IT. Finally, we address the challenges and future directions in advancing these novel therapeutic paradigms. This review aims to provide a comprehensive resource for future research dedicated to advancing innovative multi‐energy integrated photocatalytic systems in the field of cancer nanotheranostics.

## Graphical abstract

This comprehensive review summarizes recent advancements in piezo‐photocatalysts and photothermal‐photocatalysts for cancer therapy. It begins by outlining the principles of piezo‐photocatalysis and photothermal‐photocatalysis, followed by a discussion of various multi‐energy integrated photocatalysts designed for single‐, dual‐, triple‐, and tetra‐modal cancer treatment.

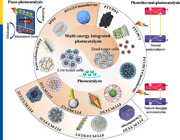

## 1 Introduction

Cancer remains one of the leading causes of death globally. Current estimates suggest that by 2030, nearly 22 million new cases will be diagnosed, leading to approximately 13 million cancer‐related deaths worldwide [1, 2]. Reactive oxygen species (ROS) are recognized as effective agents for the selective eradication of tumor cells. These species can initiate various biochemical reactions within cells, including reductions in mitochondrial membrane potential, DNA cleavage, cytoskeleton shrinkage, and chromatin aggregation, ultimately resulting in apoptosis [3, 4]. Numerous substances can generate ROS in response to external and internal stimuli, thus several therapeutic approaches have been developed for tumor treatment following this principle, including photodynamic therapy (PDT) [5], sonodynamic therapy (SDT) [6], and chemodynamic therapy (CDT) [7] etc. Collectively, these methods provide effective avenues for achieving non‐invasive tumor treatment. Among these therapies, PDT has been utilized in clinical settings due to its advantages of high tissue selectivity, minimal invasiveness, and low toxicity. However, conventional PDT is limited by the fact that most photosensitizers can only be excited by ultraviolet (UV) and visible light [8]. Besides, tumor tissues typically exhibit a hypoxic atmosphere due to distorted blood vessels, rapid cellular proliferation, and heightened metabolic activity [9]. What's worse, the treatment often leads to a significant depletion of tissue oxygen, which further reduces its therapeutic efficacy [10]. Photocatalytic therapy (PCT) leverages photocatalysts to directly oxidize water (H_2_O) molecules to generate ROS, attracting increasing attention in the field of cancer therapy. This process is independent of the concentration of dissolved oxygen or hydrogen peroxide (H_2_O_2_) in the tumor microenvironment (TME), thus facilitating the progress of PDT [11, 12].

**FIGURE 1:**
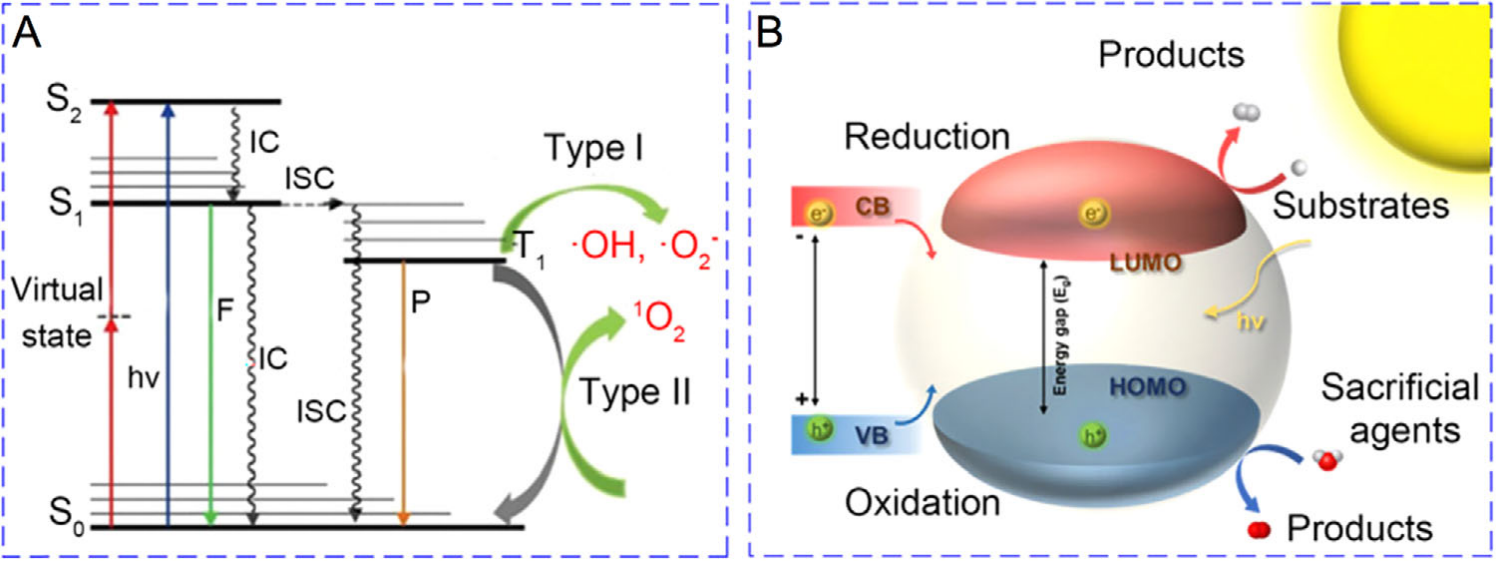
Schematic diagram of the photochemical reactions for (A) molecular organic photocatalysts and (B) semiconductors. Reproduced with permission [21]. Copyright 2024, The Royal Society of Chemistry.

Photocatalysis has long been utilized across various domains, including energy storage [13], wastewater treatment [14], and fuel production [15] etc. In recent years, its potential in biomedical applications, such as antibacterial treatment [16], tissue engineering [17], and cancer therapy [18], has gained significant interest. This surge in attention is primarily due to advancements in the structural optimization and surface modification of photocatalysts. Such improvements have enhanced the catalytic efficiency of photocatalysts, enabling substantial therapeutic effects even at low doses. Photocatalysis involves a range of photochemical processes catalyzed by semiconducting [19] or molecular organic photocatalysts [20] when exposed to suitable light irradiation. In this context, molecular organic photocatalysts are often referred to as photosensitizers. Upon exposure to light of the appropriate wavelength, these photosensitizers absorb photons, causing electrons to move from the singlet ground state (S_0_) to higher energy states, such as the singlet excited state (S_1_) or the second singlet excited state (S_2_). Both S_1_ and S_2_ are highly unstable and can return to S_0_ through internal conversion (IC). Furthermore, photosensitizers in the S_1_ state may undergo intersystem crossing (ISC) to reach the triplet excited state (T_1_). As T_1_ relaxes back to S_0_, energy is released either via phosphorescence or through photochemical reactions [21]. PDT exploits these photochemical processes through two primary mechanisms: type I and type II. In the type I mechanism, the T_1_ of photosensitizers reacts with intracellular substrates via electron transfer, leading to the formation of free radicals. These free radicals then interact with H_2_O and molecular oxygen, generating ROS such as hydroxyl radicals (·OH) and superoxide anions (O_2_^·−^) [22]. In contrast, the type II mechanism involves the direct energy transfer from T_1_ to molecular oxygen, producing singlet oxygen (^1^O_2_) (Figure 1A) [23]. Except molecular organic photocatalysts, semiconductors have gained significant attention in PCT due to their high light stability, ease of preparation, and the ability to control their morphology and structure. Semiconductor photocatalysts, especially in nanostructured forms, feature a band structure composed of a continuous range of energy levels. These bands are analogous to the highest occupied molecular orbital (HOMO) and lowest unoccupied molecular orbital (LUMO) in organic molecules and are referred to as the valence band (VB) and conduction band (CB), respectively [21]. The energy gap between these bands is known as the band gap (*E*_g_). The photocatalytic process in semiconductors can generally be described in three key steps: (1) photon absorption excites charge carriers; (2) these excited carriers move toward the surface of material; and (3) charge transfer occurs between the semiconductor and surrounding substrates, driving interfacial redox reactions (Figure 1B) [24, 25]. However, in many cases, the photoexcited charge carriers may recombine, releasing the absorbed energy. To counteract this, sacrificial agents are often used to capture the holes, thereby reducing recombination. Common sacrificial agents in physiological settings include glutathione (GSH), H_2_O, glucose, and reduced β‐nicotinamide adenine dinucleotide (NADH). In addition to generating ROS, photocatalytic processes such as H_2_O splitting [26, 27], carbon dioxide (CO_2_) reduction [28, 29], and GSH oxidation [30, 31] also show promise for therapeutic applications in cancer treatment.

Organic photosensitizers commonly used in PDT, such as chlorin e6 (Ce6), zinc phthalocyanine (ZnPc), and indocyanine green (ICG), encounter significant limitations, including issues with photobleaching, instability, and rapid systemic circulation, which hinder their broader biomedical use [32, 33]. In this context, inorganic semiconductor photocatalysts present promising alternatives for the generation of ROS under broad‐band light irradiation [34]. Semiconductors like zinc oxide (ZnO) [35, 36] and titanium dioxide (TiO_2_) [37, 38] are known for their high efficiency in ROS generation in aqueous environments. However, their relatively large *E*_g_ (3.4 eV for ZnO and 3.2 eV for TiO_2_) limit their effectiveness in PDT applications. Furthermore, as the particle size of these materials decreases to the nanoscale, the *E*_g_ tends to increase. This quantum confinement effect causes a shift in the VB and CB levels towards more positive and negative potentials, respectively, altering their photocatalytic properties. For effective PCT, the *E*_g_ of photocatalyst needs to fall within the near‐infrared (NIR, 650–1100 nm) to ensure deep tissue penetration and minimize photodamage to living organisms [39, 40]. Although some narrow‐band‐gap semiconductors can directly absorb light in the biocompatible window, most of them suffer from low NIR light conversion efficiency. To overcome this limitation, several strategies have been developed to enhance NIR‐driven photocatalysis: (1) doping wide‐band‐gap semiconductors with foreign atoms, introducing structural disorder, or creating defects; (2) combining wide‐band‐gap semiconductors with narrow‐band‐gap materials; (3) incorporating plasmonic materials such as noble metal nanoparticles or non‐metallic semiconductors into traditional semiconductors; (4) integrating upconverting luminescent materials with conventional semiconductors; (5) constructing dye‐sensitized photocatalytic systems with photosensitizers. These strategies not only enhance NIR photocatalysis but also improve the photocatalytic efficiency of semiconductor materials, thereby increasing ROS generation and optimizing tumor treatment outcomes (Table 1). For example, doping can alter the phase, morphology, size, and electronic structure of inorganic nanomaterials, leading to enhanced catalytic performance [41, 42]. Introducing vacancies or interstitials in the semiconductor structure creates localized states that trap charge carriers, improving recombination rates and boosting photocatalytic efficiency [43, 44]. The formation of heterojunctions can spatially separate photogenerated electron–hole pairs, preventing recombination and increasing photocurrent and photocatalytic activity [45, 46]. Dye sensitization can improve light absorption by semiconductors and modify the transport of photogenerated carriers, extending their lifetime and enhancing catalytic efficiency [47, 48]. Furthermore, the addition of cocatalysts such as platinum (Pt) [49], palladium (Pd) [50], or nickel (Ni) [51] can accelerate reduction and oxidation reactions, promoting faster charge transfer. Finally, using sacrificial agents to donate electrons or accept holes can further increase the availability of charge carriers for photocatalytic reactions [52, 53].

**TABLE 1:** A comparison of the key strategies for enhancing NIR‐driven photocatalysis.

Strategies	Mechanism/examples	Advantages	Limitations
Narrow‐bandgap semiconductors	Black phosphorus, Cu_2_O, WO_3−_ *_x_*, Ti_3_C_2_ MXenes	Direct NIR absorption, cost‐effective	Rapid charge recombination, photocorrosion
Defect engineering	Oxygen vacancies, sulfur vacancies, or doping	Introduces mid‐gap states for NIR absorption	May reduce crystallinity or stability
Heterojunction engineering	Z‐scheme, Schottky junctions, or Type‐II band alignment	Enhances charge separation, broad‐spectrum response	Complex interface design
NIR‐sensitive organic dyes	Dyes (e.g. cyanines, phthalocyanines) absorb NIR and transfer energy to semiconductors	High molar absorptivity, tunable structures	Photodegradation, poor stability
LSPR	Noble metal nanoparticles (e.g. Au, Ag) absorb NIR light via LSPR, generating hot electrons for catalysis	Strong NIR absorption, tunable resonance	High cost, electron–hole recombination
Upconversion materials	Lanthanide‐doped nanoparticles (e.g. NaYF_4_:Yb^3+^/Tm^3+^) convert NIR to UV/visible light for wide‐bandgap photocatalysts	Broad NIR absorption, stable output	Low quantum yield, complex synthesis
Sacrificial agents	Hole scavengers (electron donors), electron acceptors (hole generators)	Enhances charge separation and reduces recombination, tunable selectivity for specific reactions, compensation for weak NIR light absorption	Not sustainable, potential toxicity, requires optimization
Photothermal synergy	NIR‐induced heating (e.g. carbon materials) boosts catalytic kinetics	Improves reaction rates, utilizes waste heat	Temperature control challenges

In addition to the strategies previously discussed, applying an external field emerges as a versatile and controllable method for enhancing catalytic activity without modifying the fundamental properties of the semiconductor material. A novel and rapidly evolving area of research, multi‐energy integrated photocatalysis, has recently gained prominence. This approach utilizes the synergistic combination of multiple energy sources such as sound [54, 55], electricity [56, 57], heat [58, 59], and magnetism [60, 61] to improve photocatalytic performance. The integration of these energies offers several key benefits, including overcoming *E*_g_ limitations, improving charge separation and transport, enhancing reactant adsorption and activation, and increasing the overall efficiency of photocatalytic reactions. Moreover, this strategy significantly boosts the stability and longevity of photocatalysts, accelerates reaction rates, and enhances reaction kinetics. The method also showcases versatility across various fields, improves selectivity and control over the reactions, and can reduce costs, thus facilitating practical implementation in real‐world applications [62, 63]. These advantages make multi‐energy integrated photocatalysis an attractive option for a wide range of potential uses. Currently, multi‐energy integrated photocatalysis is generally divided into four primary categories: piezo‐photocatalysis, photoelectrocatalysis, photothermal‐photocatalysis, and magnetic‐field‐enhanced photocatalysis. Among these, piezo‐photocatalysis and photothermal‐photocatalysis have attracted significant attention in biomedicine, particularly for their promise in highly efficient cancer treatments. Piezoelectric semiconductor materials, such as ZnO and bismuth (Bi)‐based layered compounds, exhibit not only piezoelectric effects but also piezo‐photoelectronic properties when exposed to light [64, 65]. These materials, acting as piezo‐photocatalysts, generate photogenerated electron–hole pairs under the combined influence of light and mechanical stress. The application of external mechanical strain induces a piezoelectric field, which further promotes the separation of charge carriers, facilitating the piezo‐photocatalytic reaction [66]. On the other hand, photothermal‐photocatalysis represents an innovative fusion of light and thermal energy within catalytic systems, enhancing the overall reaction efficiency. By incorporating photothermal properties, these photocatalysts can raise the local temperature, accelerating reaction kinetics. This makes photothermal‐photocatalysis especially attractive for biomedical applications, where precise control over reaction conditions is crucial for optimal curative outcomes [67, 68].

Beyond the therapeutic applications, diagnostic imaging plays a crucial role in oncology by enabling precise tumor localization and real‐time treatment monitoring. Techniques such as photoacoustic imaging (PAI) [69], fluorescence imaging (FLI) [70], and computed tomography (CT) [71] have gained widespread attention for their unique advantages. PAI combines deep tissue penetration with high resolution and functional imaging, enabling precise visualization of tumor structures and molecular activity without the use of ionizing radiation [72]. FLI provides ultra‐sensitive, real‐time imaging at the molecular and cellular levels, offering exceptional multiplexing capabilities for dynamic tracking of biological processes with high resolution [73]. CT excels in 3D anatomical mapping of deep tissues, making it ideal for precisely targeting deep or diffuse tumors while overcoming the penetration limitations of optical methods [74]. Multifunctional photocatalysts demonstrate exceptional potential for multimodal cancer imaging by synergistically combining tailored material properties with efficient energy conversion mechanisms. For PAI, nanomaterials including copper sulfide (CuS) [75] and black phosphorus (BP) [76] leverage their strong NIR absorption and exceptional photothermal conversion efficiency (PCE) to simultaneously enable photothermal therapy (PTT) and photoacoustic signal generation. These systems integrate seamlessly with FLI through either intrinsic fluorescent performance, exemplified by carbon dots (CDs) [77] and porphyrin derivatives [78], or Förster resonance energy transfer (FRET) processes [79], allowing dynamic tracking of ROS production and therapeutic response. Complementing these modalities, high‐atomic‐number (high‐Z) elements such as bismuth (Bi) [80], tungsten (W) [81], and gold (Au) [82] provide enhanced X‐ray attenuation for precise CT, facilitating accurate deep‐tissue tumor localization and image‐guided therapeutic interventions. Notably, these imaging capabilities are intrinsic to the design of photocatalysts, allowing for seamless theranostic integration without the need for external contrast agents. By combining multiscale tumor visualization with treatment monitoring and outcome assessment, multi‐energy photocatalysts present a powerful platform for precision oncology.

**SCHEME 1:**
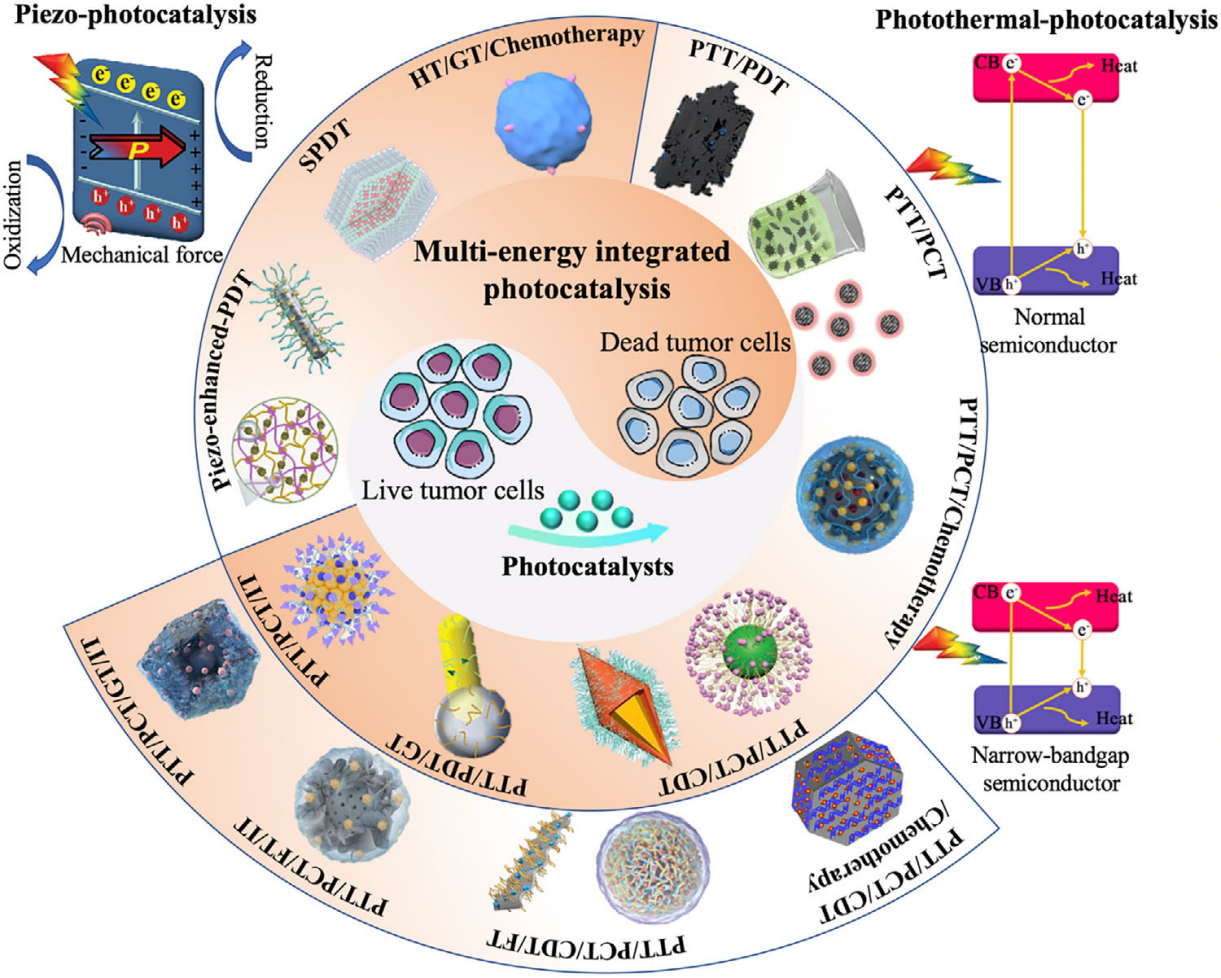
Schematic illustration of various piezo‐photocatalysts and photothermal‐photocatalysts for cancer treatment.

Up to now, research on multi‐energy integrated photocatalysis is still in its early stages. In light of the current global energy crisis, several studies have reviewed the application of multi‐energy integrated photocatalysis in the fields of energy [83, 84, 85, 86, 87], environment [54, 59, 65, 88, 89], organic synthesis [90], and pollutants degradation [65], but reports on their biomedical applications are scarce. In this work, we for the first time present a comprehensive summary of emerging multi‐energy integrated photocatalysis for cancer treatment (Scheme 1). Unlike single‐modal therapies, multi‐modal approaches offer enhanced therapeutic outcomes by targeting cancer from multiple angles, including controlling local tumor growth, eliminating systemic micrometastases, and overcoming tumor heterogeneity through synergistic interactions. This integrated strategy not only improves treatment efficacy but also reduces the risks of resistance, enables dose optimization, and lowers toxicity, all while maintaining or even improving survival rates. Despite the increased complexity in coordination, the proven benefits of multi‐modal therapies have established them as the standard of care for many aggressive or advanced cancers. The discussion begins with an introduction to the principle of piezo‐photocatalysis, followed by an exploration of its potential in piezo‐enhanced cancer treatments such as piezoelectric‐enhanced single‐modal PDT, dual‐modal sono‐photodynamic therapy (SPDT), and triple‐modal hydrodynamic therapy (HT)/gas therapy (GT)/chemotherapy. We then paraphrase photothermal‐photocatalysis and summarize the incorporation of additional treatment modalities to obtain dual‐modal PTT/PCT and PTT/PDT, triple‐modal PTT/PCT/chemotherapy, PTT/PCT/CDT, PTT/PCT/GT, and PTT/PCT/immunotherapy (IT), and tetral‐modal PTT/PCT/CDT/chemotherapy, PTT/PCT/CDT/ferroptosis therapy (FT), PTT/PCT/FT/IT, and PTT/PCT/GT/IT. Finally, we address the potential challenges and future perspectives associated with these novel cancer therapeutic paradigms.

## 2 Piezo‐Photocatalysis

### 2.1 Principle of Piezo‐Photocatalysis

In 2006, Wang et al. first demonstrated the piezoelectric nanogenerator based on ZnO nanowire arrays, which established a paradigm for converting mechanical energy into electrical energy at the nanoscale [91]. This breakthrough has since inspired extensive research on mechano‐energy conversion, particularly in developing hybrid systems that transform mechanical stimuli into electrical/chemical energy to drive photocatalytic processes. Piezoelectric materials, with their non‐centrosymmetric structures, allow mechanical strain to induce polarization. When stress is applied, the polarity of the electric field shifts in response to the direction of the strain, leading to a displacement of positive and negative charge centers. This displacement generates an internal electric field, which in turn accelerates catalytic reactions [92, 93]. Furthermore, certain piezoelectric semiconductors exhibit both piezoelectric and piezo‐photoelectric effects under light exposure, classifying them as piezo‐photocatalysts. This combination leverages the benefits of both piezocatalysis‐such as strong catalytic activity, high efficiency, excellent compatibility, long‐lasting catalytic effects, and low cost‐and photocatalysis, which offers a straightforward process, minimal secondary pollution, strong oxidation power, and cost‐effectiveness. Together, these advantages enable the development of highly efficient piezo‐photocatalytic system [90, 94, 95]. Under the simultaneous influence of light and mechanical stress, piezo‐photocatalysts generate electron–hole pairs while the piezoelectric field enhances carrier separation. These piezo‐photocatalysts are typically composite structures, combining piezoelectric materials with semiconductors, often in core–shell nanostructures. In such systems, the core consists of piezoelectric materials like ZnO, barium titanate (BaTiO_3_), or sodium niobate (NaNbO_3_), while the shell is made of visible‐light photocatalysts such as CuS, ferrous sulfide (FeS), or silver oxide (Ag_2_O). Some piezoelectric materials with narrow *E*_g_ also display intrinsic photocatalytic properties, allowing them to simultaneously generate both piezopotential and photo‐induced charge carriers within a single material [54, 96]. Efficient charge carrier separation and transport are critical for optimizing catalytic performance, especially in the context of piezo‐photocatalysis. In a landmark study by Li et al. [97], a BaTiO_3_/Ag_2_O hybrid catalyst was developed to evaluate its efficacy in degrading the organic dye rhodamine B (RhB) under the combined effects of ultrasonic excitation and UV light exposure. During periodic ultrasonic excitation, BaTiO_3_ nanocrystals spontaneously generate a polarization potential, creating an alternating electric field. This field facilitates the separation of photo‐generated carriers in Ag_2_O, driving them to opposite sides of the BaTiO_3_ nanocubes. This mechanism effectively suppresses carrier recombination, thereby enhancing both the photocatalytic activity and the cyclic stability of the hybrid catalyst. In addition to BaTiO_3_/Ag_2_O, other hybrid piezo‐photocatalytic systems, such as ZnO/CuS [98], BaTiO_3_/cupric oxide (CuO) [99], bismuth ferrite (BiFeO_3_)/covalent organic frameworks (COF) [100], have demonstrated promising results. These materials show improved photocatalytic performance under both UV and visible light, positioning them as strong candidates for applications in H_2_O splitting and pollutant degradation. However, despite the growing potential of piezo‐photocatalysts, their use in solid tumor treatment remains constrained. This limitation is primarily due to the insufficient performance of these catalysts in the NIR light range, as their *E*_g_ often exceed 2.9 eV, much higher than the photon energy of 808 nm NIR light. As a result, these catalysts are poorly equipped to absorb NIR light, leading to low ROS production and limiting their efficacy in phototherapy.

Therefore, there is an urgent need for the development of novel piezo‐photosensitizers that feature narrow *E*_g_, rapid carrier separation, and strong piezoelectric properties. Such materials could significantly enhance tumor treatment outcomes through piezo‐enhanced PDT or PCT. The inherent complexity, diversity, and heterogeneity of tumors make single‐modal treatment approaches often ineffective. To address these challenges, researchers have actively pursued the integration of various therapeutic components within a single nanoplatform through rational design strategies. This combined method seeks to achieve superadditive anticancer efficacy while addressing the limitations associated with individual treatment modalities. In this section, we present the recent piezo‐photocatalysts for single‐modal, dual‐modal, and triple‐modal cancer therapy.

### 2.2 Piezo‐Photocatalysts for Cancer Therapy

#### 2.2.1 Piezo‐Photocatalysts for Single‐Modal Cancer Therapy

Localized surface plasmon resonance (LSPR) is a phenomenon that occurs in metallic nanoparticles, where conduction electrons oscillate collectively in response to an external electromagnetic field, particularly light. By leveraging the unique properties of metallic nanoparticles, LSPR enables advanced applications in sensing, imaging, and therapeutic techniques [101, 102]. As for photocatalysis, LSPR is able to enhance light wavelength utilization by improving light absorption, generating charge carriers, facilitating hot electron injection, increasing reaction rates, broadening the absorption spectrum, and improving light scattering. These effects collectively contribute to more efficient photocatalytic processes, making plasmonic materials valuable in advancing photocatalytic technologies [103, 104, 105].

**FIGURE 2:**
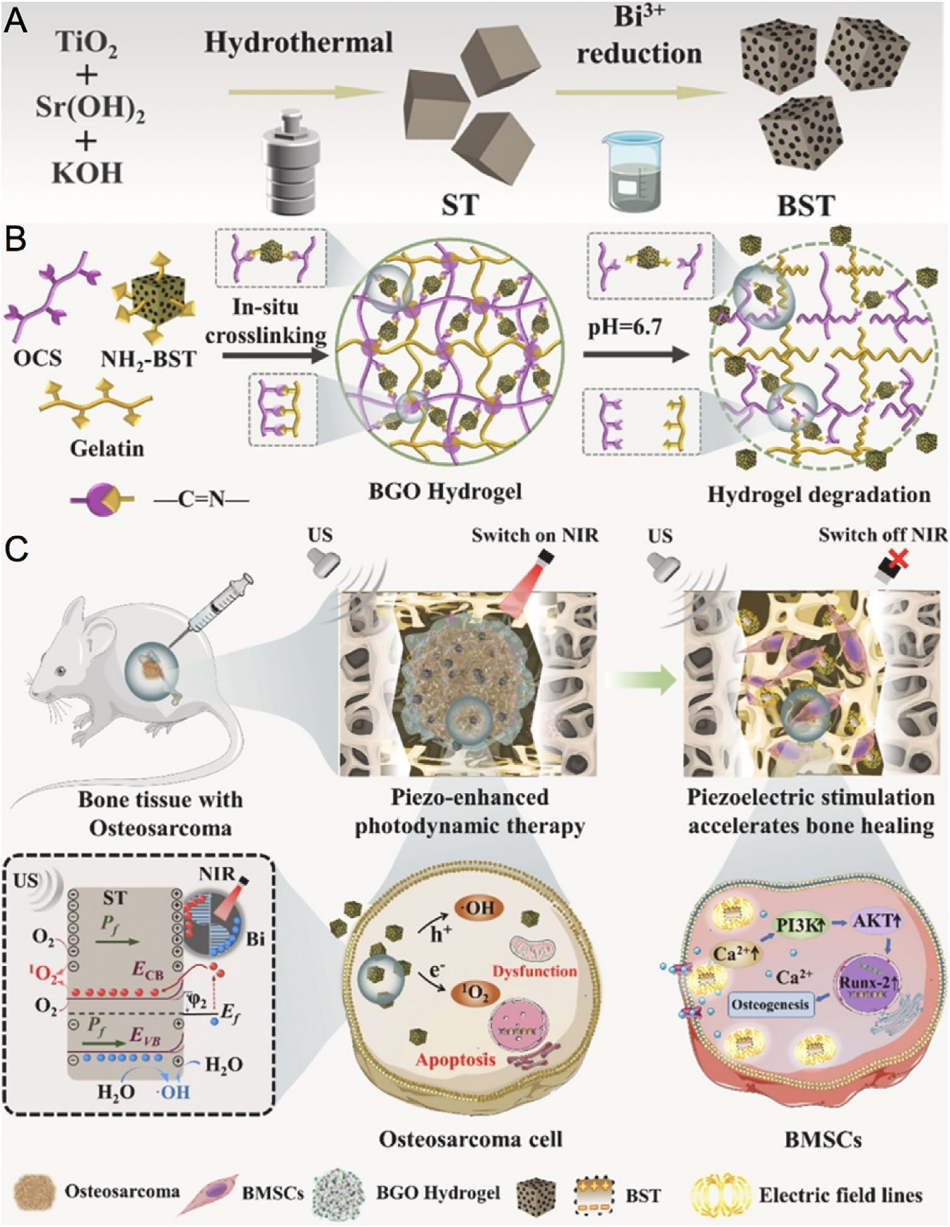
(A) The scheme of synthetic procedure of BST nanoparticles. Piezoelectric field‐driven fast charge separation coupling with surface plasmon resonance enhanced near‐infrared photodynamic therapy for antiosteosarcoma and osteogenesis (B) The amino group of BST and gelatin, as well as the aldehyde group of oxidized chondroitin sulfate, were utilized for crosslinking via Schiff base bonds to prepare a BST/Gelatin/OCS nanocomposite injectable hydrogel (BGO hydrogel). Importantly, the dynamically covalent (Schiff base bond) cross‐linked BGO hydrogel continuously released BST nanoparticles in response to the tumor microenvironment (pH <6.7). (C) Benefiting from piezoelectric field‐driven fast charge separation coupling with surface plasmon resonance, BST nanoparticles demonstrate a remarkable capability to efficiently generate ROS in response to NIR, thereby effectively eliminating tumor cells. Furthermore, in the NIR switch‐off stage, endocytosed BST nanoparticles coupled with US treatment generate intracellular electrical stimulation to promote osteogenic differentiation and accelerate bone healing in vitro. Reproduced with permission [106]. Copyright 2024, Elsevier B.V.

Xiao et al. [106] developed a Bi/SrTiO_3_ (BST) nanoheterostructure and embedded it into an injectable biopolymer hydrogel to simultaneously target osteosarcoma and promote osteogenesis. Strontium titanate (SrTiO_3_, denoted as ST) nanoparticles were synthesized through a hydrothermal method, followed by sodium borohydride (NaBH_4_) treatment to deposit Bi nanoparticles and form BST. These BST nanoparticles were then incorporated into a gelatin and oxidized chondroitin sulfate (OCS) matrix, crosslinked via Schiff base bonds, to create a BST/Gelatin/OCS (BGO) hydrogel. This hydrogel dynamically released BST nanoparticles in response to the acidic TME (pH <6.7). The BST nanoheterojunction, thanks to its LSPR and narrow *E*_g_, demonstrated responsiveness to light across the UV to NIR spectrum. Upon NIR irradiation, the Bi nanoparticles induced collective oscillation of surface electrons, generating high‐energy hot electrons that overcame the Schottky barrier and transferred to the CB of ST. The resultant holes triggered oxidation reactions with donor species, while external ultrasonic vibrations generated a piezoelectric field within the ST nanoparticles. This piezoelectric effect bended the CB and VB downward, further enhancing the generation of ROS, such as ·OH and ^1^O_2_, within BST. The reduction of the Schottky barrier prevented the recombination of high‐energy hot carriers induced by LSPR, thus boosting free radical production. In vivo studies showed that the BST nanoparticles effectively treated human malignant patient‐derived xenografts (PDX) and tibial orthotopic osteosarcoma models through piezoelectric‐enhanced PDT. Moreover, after NIR irradiation ceased, endocytosed BST nanoparticles, combined with ultrasound (US) treatment, induced intracellular electrical stimulation, promoting osteogenic differentiation and accelerating bone healing in vitro. Overall, this study demonstrates the successful integration of plasma metal/piezoelectric heterojunctions in biopolymer hydrogels for effective anti‐osteosarcoma therapy and accelerated bone regeneration via piezo‐enhanced PDT and electrically‐triggered osteogenesis (Figure 2).

**FIGURE 3:**
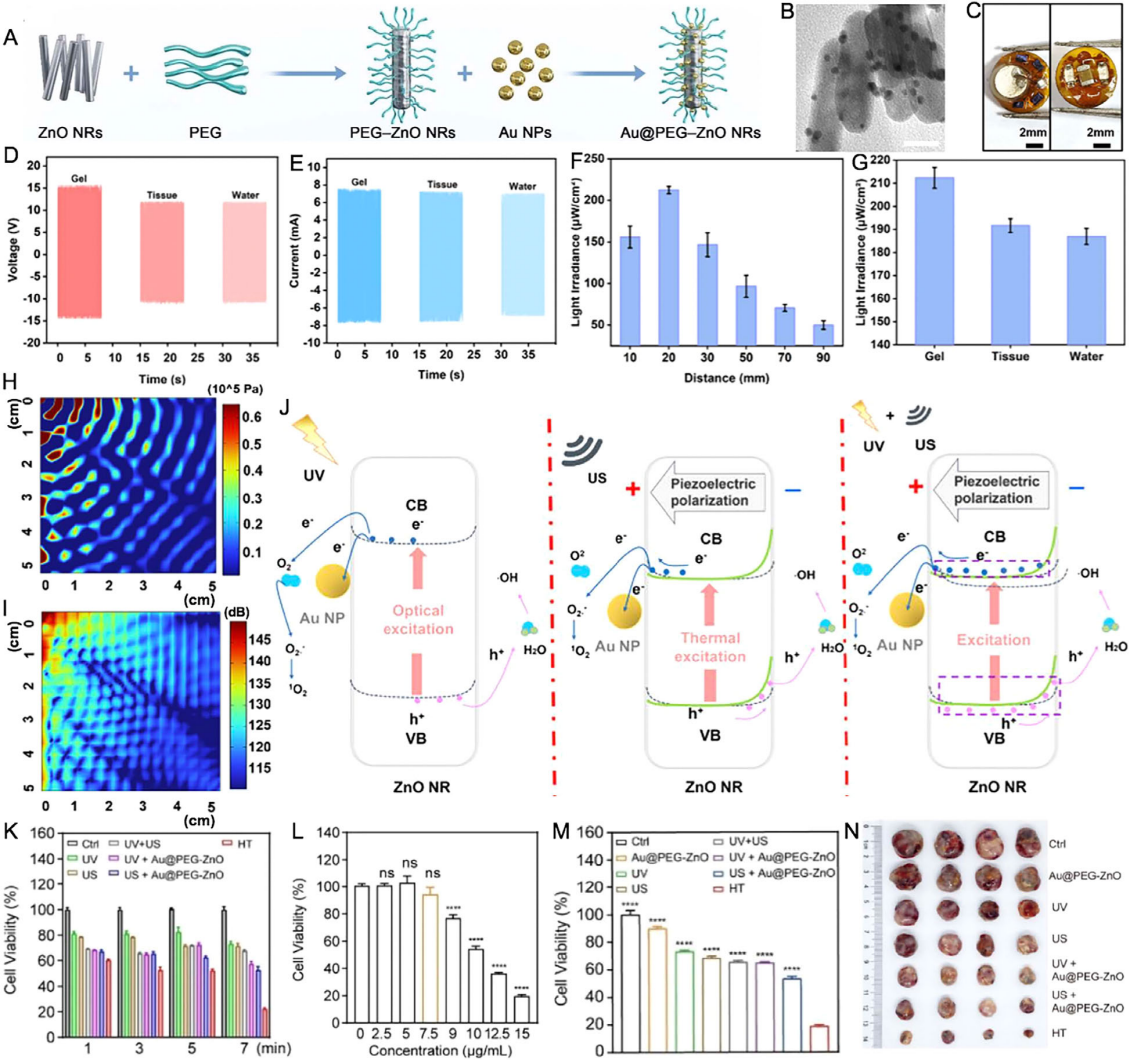
(A) Synthesis process of the nanocomposites. (B) Transmission electron microscopy (TEM) image of Au@PEG‐ZnO nanocomposites with scale bar of 20 nm. (C) Appearance of the therapeutic dot. (D) and (E) Output voltage and current in gel, tissue, and water. (F) Light illumination power of the device at different depths. (G) Light illumination power of the device in different media. (H) Simulation of sound pressure at varying depths. (I) Simulation of sound pressure levels at different depths. (J) Mechanism of ROS generation by Au@PEG‐ZnO nanocomposites under UV, US, and UV + US conditions. (K) Screening of optimal treatment time for therapeutic dot in PPDT. (L) Cell viability assessed by the CCK‐8 assay for varying concentrations of Au@PEG‐ZnO nanocomposites. (M) Cellular activity of different treatment groups under optimal parameters (7.5 µg mL^−1^ Au@PEG‐ZnO, 7 min). (N) Photographs of tumors collected from each group. Reproduced with permission [107]. Copyright 2024, Elsevier Ltd.

Han et al. [107] introduced a novel strategy that enhanced PDT for tumor suppression through the use of piezopotential‐driven ROS generation. This approach employed a wireless, battery‐free device that integrated Au nanoparticles, ZnO nanorods (NRs), and hydrophilic polyethylene glycol (PEG) (Figure 3A). As illustrated in Figure 3B, Au nanoparticles, with an average diameter of 3.8 nm, were loaded onto the surface of ZnO NRs, which had a diameter of about 18.9 nm and a length of roughly 71.3 nm. The therapeutic device, encased in biocompatible polydimethylsiloxane (PDMS), was safe for implantation. Figure 3C showed the complete device setup, which included a wireless power module, a µLED light source, and a control system. US energy was harnessed to generate electricity, which powered two 365 nm µLEDs used for treatment. Figure 3D,E displayed the piezoelectric output of device across various media: 14.2 V and 7.55 mA in medical gel, 11.73 V and 7.29 mA in pork tissue, and 11.6 V and 6.76 mA in H_2_O (US frequency: 500 kHz, power: 2 W, distance: 2 cm). The performance of the light source powered by the µLEDs was also evaluated, with light output measurements at varying distances of 10, 20, 30, 50, 70, and 90 mm showing values of 155.83, 212.23, 146.67, 96.6, 70.67, and 49.9 µW/cm^2^, respectively (Figure 3F,G). The US could penetrate up to 5 cm, demonstrating the reliability and versatility of the device (Figure 3H,I). The wireless control of the device was enabled through US activation, utilizing the piezoelectric effect to deliver light‐based treatment doses (Figure 3J). When ZnO was exposed to both US and UV light, the generation of electron–hole pairs was significantly enhanced through thermal and photoexcitation, leading to the abundant production of ROS in H_2_O. Simultaneously, the piezoelectric field induced by US radiation along the ZnO NRs caused charge carriers to drift, with electrons and holes migrating to opposite sides to generate positive and negative potentials, respectively. The positive piezoelectric potential reduced the bending of the CB, lowering the barrier for electron‐involved ROS generation reactions. Conversely, the negative pressure enhanced the bending of the VB edge, facilitating the transfer of energy to the holes for ROS generation. Furthermore, the lowered piezoelectric barrier enabled efficient electron migration from the CB of ZnO NRs to Au nanoparticles, suppressing electron–hole recombination. This unique mechanism significantly boosted ROS production, thereby improving the therapeutic efficacy of the system. Both in vitro and in vivo studies, including a preclinical mouse lung cancer model, demonstrated superior therapeutic outcomes when compared to individual PDT and SDT treatments (Figure 3K–N).

#### 2.2.2 Piezo‐Photocatalysts for Dual‐Modal Therapy

Rare earth upconversion nanoparticles (UCNPs) are a type of anti‐Stokes law luminescent material that emits visible light when excited by NIR radiation. These nanoparticles convert long‐wavelength radiation into short‐wavelength radiation through a multiphoton mechanism. Notably, the fluorescence of UCNPs is stable and flicker‐free, enhancing their suitability for both in vivo and in vitro follow‐up therapies [108, 109, 110]. Compared to tetragonal UCNPs, hexagonal UCNPs demonstrate higher upconversion efficiency, longer luminescence lifetimes, and improved chemical stability [111]. In most cases involving upconversion nanomaterials, β‐NaYF_4_ is selected as the most efficient host material, particularly when combined with suitable dopant lanthanide ions that serve as activators (e.g. erbium ions (Er^3+^), thulium ions (Tm^3+^), and holmium ions (Ho^3+^)) or sensitizers (e.g. ytterbium ions (Yb^3+^)), depending on the application [112, 113]. Recent years have seen an increasing focus on integrating this upconversion process with semiconductor materials such as TiO_2_ [114], ZnO [115], cadmium sulfide (CdS) [116], zinc cadmium sulfide (Zn*_x_* Cd_1−_ *_x_* S) [117], CuS [118], and so on. In this regard, UCNPs significantly improve the efficiency and effectiveness of photocatalytic processes by harnessing low‐energy light and providing enhanced excitation conditions, making them a promising tool for various ROS‐based applications.

**FIGURE 4:**
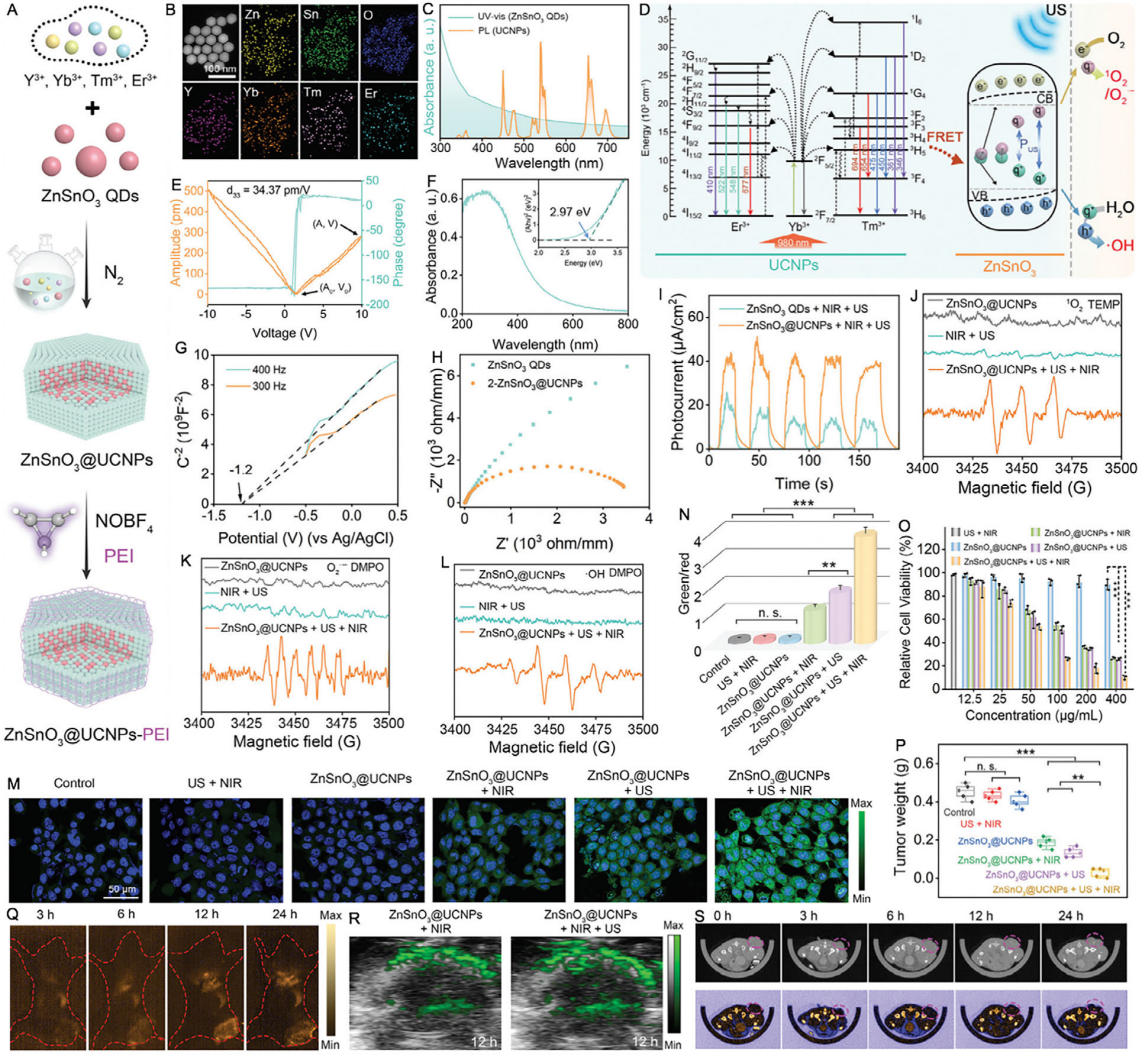
(A) Schematic depiction of the preparation process of ZnSnO_3_@UCNPs. (B) TEM, high‐angle annular dark field scanning transmission electron microscopy (HAADF‐STEM), and corresponding elemental mapping images of ZnSnO_3_@UCNPs synthesized at different temperatures and times. (C) UCL spectra of the UCNPs (orange) and the UV–vis absorption spectra (green) of the ZnSnO_3_ QDs. (D) Schematic illustration of energy transfer from UCNPs to QDs and subsequent ROS generation mechanism. (E) In‐plane amplitude, amplitude‐voltage (orange), and phase‐voltage (green) curves of ZnSnO_3_@UCNPs. (F) UV–vis absorption spectrum and Kubelka–Munk plot of ZnSnO_3_ QDs. (G) Mott–Schottky plots of ZnSnO_3_ QDs. (H) Electrochemical impedance spectra and (I) ultrasonic current test of ZnSnO_3_ QDs and ZnSnO_3_@UCNPs under the continuous NIR (0.4 W cm^−2^) and US (40 kHz). (J–L) Electron spin resonance (ESR) spectra of O_2_^·−^, ^1^O_2_, and ·OH under different conditions. (M) Confocal laser scanning microscopy (CLSM) images and flow cytometry analysis of the associated mean fluorescence intensity of intracellular ROS level under various conditions. (N) JC‐1 staining of 4T1 cells in various treatments is shown in quantitative analysis. Statistical analysis was conducted via one‐way ANOVA with a Bonferroni post hoc test. *p < 0.05, **p < 0.01, ***p < 0.001. Data are presented as mean ± SD (*n* = 3). (O) Cytotoxicity of 4T1 cells after treated with different formulations. (Statistical analysis was performed via one‐way analysis of variance with a Bonferroni post hoc test. Data are presented as mean ± SD (*n* = 3), ***p < 0.001.). (P) The mean tumor weight of 4T1 tumor‐bearing mice in each group after 14 d of treatment. Data are expressed as mean ± SD (*n* = 5). Statistical significance was assessed by unpaired Student's two‐sided *t*‐test. *p < 0.01, **p < 0.005, ***p < 0.001. (Q) NIR‐II images of 4T1 tumor‐bearing mice irradiated by 980 nm laser at different times after intravenous injection at ZnSnO_3_@UCNPs. (R) Photoacoustic images of tumor site after intravenous administration for 12 h and after US stimulation (1 min). (S) Transverse view of tumor area after intravenous injection at different time intervals ZnSnO_3_@UCNPs (200 µg mL^−1^). Reproduced with permission [119]. Copyright 2024, Wiley‐VCH GmbH.

In contrast to standalone SDT or PDT, SPDT offers superior tumor penetration, enhanced ROS generation, better hypoxia tolerance, and reduced resistance. This makes it a promising strategy for treating deep‐seated, aggressive, or hypoxic tumors while minimizing side effects. Zhang et al. [119] employed a one‐pot thermal decomposition method to encapsulate zinc stannate quantum dots (ZnSnO_3_ QDs) with hexagonal UCNPs, achieving superior piezo‐photoelectronic performance for enhanced SPDT (Figure 4A). TEM images illustrated the uniformity of ZnSnO_3_@UCNPs, averaging 44 nm in size, obtained under reaction conditions of 300°C for 80 min, while elemental mapping confirmed the presence of each constituent (Figure 4B). The QDs exhibited broad UV and visible light absorption spectra, aligning with the emission wavelengths of designed Er^3+^/Tm^3+^ sensitized UCNPs, facilitating FRET with an efficiency as high as 80.30% (Figure 4C). As illustrated in Figure 4D, the ZnSnO_3_@UCNPs exhibited three key piezoelectric and photoelectronic effects including photoexcitation, piezoelectric, and semiconductor properties. Figure 4E demonstrated the conventional piezoelectric capabilities, with the calculated longitudinal piezoelectric coefficient (*d*_33_) value (34.37 pm V^−1^) indicating significant piezoelectric potential generation by force‐induced deformation in ZnSnO_3_@UCNPs. The band structure was subsequently analyzed to elucidate the formation principle of SPDT. As shown in Figure 4F, the ultraviolet‐visible (UV–vis) diffuse reflectance spectrum of ZnSnO_3_ QDs revealed an *E*_g_ of 2.97 eV. Electrochemical analysis depicted a flat‐band potential *E*_fb_ (vs Ag/AgCl) of ZnSnO_3_ QDs at about −1.2 V relative to the Ag/AgCl electrode, and approximately −0.588 V relative to the normal hydrogen electrode (NHE), thus establishing a VB potential of 2.382 V (vs NHE) (Figure 4G). The smaller radius arc of ZnSnO_3_@UCNPs suggested that the formation of heterogeneous structures reduced resistance and facilitated faster charge pair transfer (Figure 4H). Furthermore, ZnSnO_3_@UCNPs exhibited a higher transient current (0.42 mA cm^−2^), demonstrating that the piezoelectric photoelectronic effect enhanced charge separation and transfer (Figure 4I). Upon NIR and US irradiation, O_2_ → O_2_^·−^/^1^O_2_ and H_2_O → ·OH transformations occurred on the surface of ZnSnO_3_@UCNPs, respectively (Figure 4J‐L). Intracellular ROS levels were assessed using dichlorodihydrofluorescein diacetate (DCFH‐DA), with the ZnSnO_3_@UCNPs + NIR + US group exhibiting the brightest green fluorescence, indicating its superior ROS generation capability (Figure 4M). Subsequently, the JC‐1 probe was employed to evaluate mitochondrial function. Semiquantitative analysis of the ratio of green monomers to red polymers using ImageJ software revealed that the ZnSnO_3_@UCNPs + NIR + US group exhibited a 3.2‐fold higher ratio compared to the ZnSnO_3_@UCNPs + NIR group and a 2.1‐fold higher ratio compared to the ZnSnO_3_@UCNPs + US group, confirming the enhanced cytotoxicity of the ZnSnO_3_@UCNPs + NIR + US group (Figure 4N). As expected, the survival rate was 26.47% at 100 µg mL^−1^ in the ZnSnO_3_@UCNPs + NIR + US group, confirming the highly effective therapeutic outcome of SPDT under combined NIR and US treatment (Figure 4O). In vivo therapeutic results consistently demonstrated that the ZnSnO_3_@UCNPs + US + NIR group exhibited the highest tumor inhibition rate (98.8%) (Figure 4P). In addition, ZnSnO_3_@UCNPs served as contrast agents for in vivo upconversion luminescence (UCL) imaging, CT, and PAI, supporting integrated diagnostic and therapeutic applications (Figure 4Q–S).

In piezoelectric‐enhanced photocatalytic cancer therapy, the *d*_33_ plays a primary role by governing charge generation under mechanical stress, directly amplifying the production of ROS and facilitating deeper tumor penetration. On the other hand, the shear piezoelectric coefficient (*d*_41_) fine‐tunes the distribution of the electric field in anisotropic materials and enables flexible integration into devices targeting superficial tumors. While *d*_33_ primarily drives the core therapeutic effects through a robust mechano‐catalytic synergy, *d*_41_ provides complementary benefits in spatial field modulation and hybrid system design. Optimizing both coefficients could enable precise control over piezoelectric potentials, enhancing tumor‐specific therapy [120, 121].

#### 2.2.3 Piezo‐Photocatalysts for Triple‐Modal Cancer Therapy

Chemiluminescence is defined by a chemical reaction that produces light from fluorescent substances without requiring an external light source. A classic example is the oxidation of luminol, which is one of the most widely used luminescent materials in electrochemical luminescence detection due to its high luminescent efficiency, low cost, and excellent biocompatibility [122]. When combined with an oxidizing agent such as H_2_O_2_ or metal ions, luminol generates an excited‐state intermediate that is able to release energy in the form of photons as it returns to its ground state, resulting in the emission of visible light [123, 124]. The integration of chemiluminescence and photocatalysis presents a promising avenue for innovative research and practical applications, particularly in the biomedical fields. The visible light produced by chemiluminescent reactions can effectively excite photocatalysts like TiO_2_ or ZnO [125, 126]. When these catalysts absorb the emitted light, they generate electron–hole pairs that can participate in photocatalytic reactions. Chemiluminescent reactions typically occur at room temperature and involve simple experimental setups, making them attractive for practical applications. In specific imaging and PDT scenarios, the light produced by chemiluminescence can activate photosensitizers, leading to the generation of ROS that selectively target and kill tumor cells [127, 128]. This reduces the reliance on complex lighting equipment, thereby simplifying the setup and lowering costs. Furthermore, the ability to conduct reactions under low‐light conditions enhances the applicability of these methods across various environments. By leveraging the unique properties of chemiluminescence, researchers can develop more efficient and adaptable photocatalytic systems to address a range of challenges [129, 130].

**FIGURE 5:**
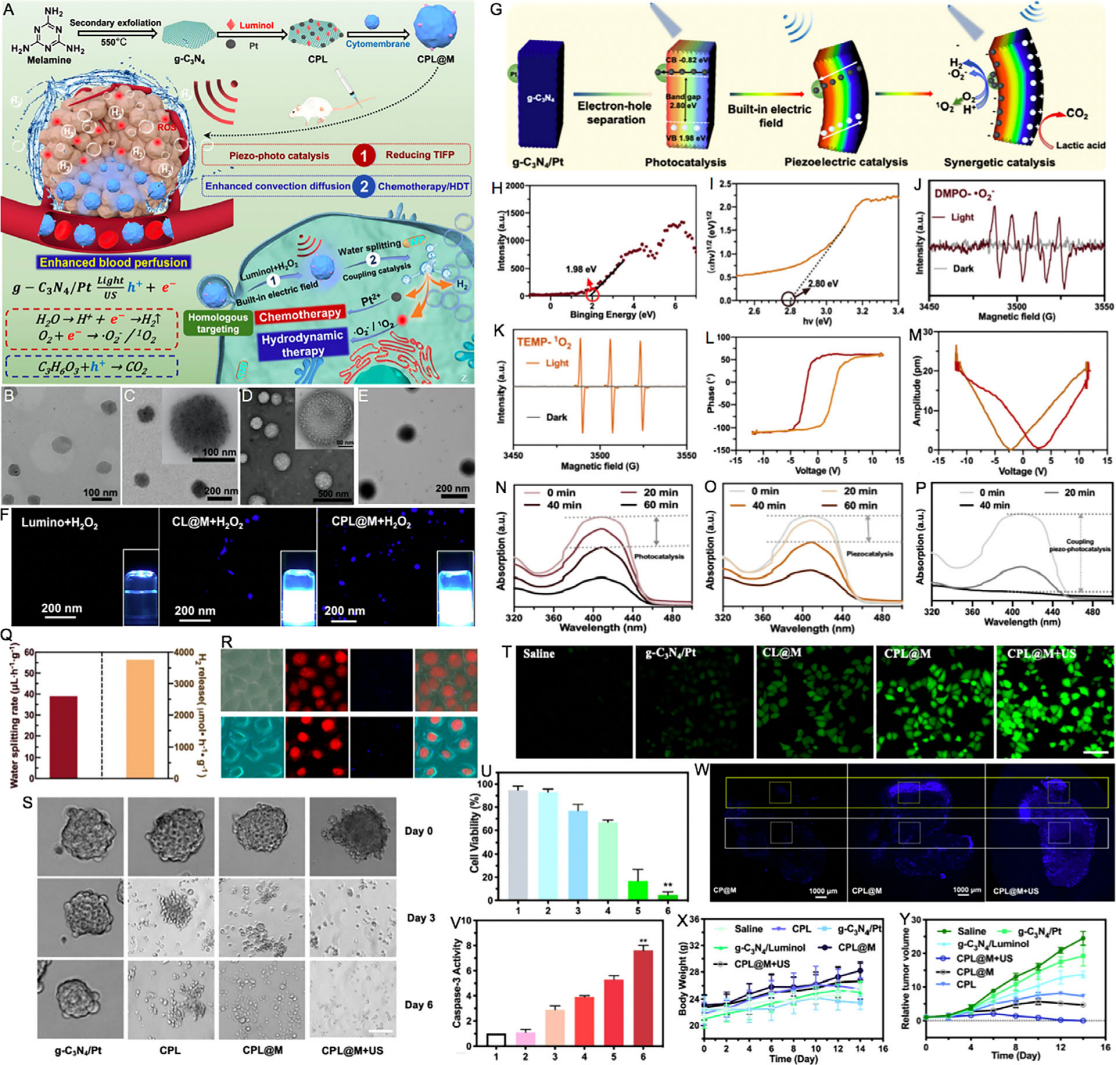
(A) Schematic illustration of nano‐lymphatic vessels for mechanism of reducing TIFP, deep penetration and HT. TEM images of (B) g‐C_3_N_4_, (C) CPL, (D) cytomembrane (CM), and (E) CPL@M. (F) Fluorescent images of Luminol + H_2_O_2_, CL@M + H_2_O_2_ and CPL@M + H_2_O_2_. (G) Schematic illustration of the g‐C_3_N_4_/Pt‐mediated water splitting process under the photo‐piezoelectric coupling catalysis. (H) Band structure alignments of g‐C_3_N_4_/Pt. (I) plots of transformed Kubelka–Munk function versus photon energy of g‐C_3_N_4_/Pt. ESR spectra of (J) 5,5‐dimethyl‐1‐pyrroline N‐oxide (DMPO)/O_2_^·−^ and (K) 2,2,6,6‐tetramethylpiperidine (TEMP)/^1^O_2_ in the presence of CPL@M. (L, M) Hysteresis loop of CPL@M. (N–P) ROS production of CPL@M detected by DCFHDA under the photocatalysis, piezocatalysis and coupling piezo‐photocatalysis in different time points. (Q) Mole rates of H_2_ release and volume rates of coupling piezo‐photocatalytic water splitting in LA solution (1 %). (R) Fluorescence dyeing of HeLa cells treated with CPL or CPL@M at 1 h for testing homologous‐targeting ability of cytomembrane. (S) Growth of 3D cell spheres with different treatments at 0, 3, and 6 days with the medium of 2 mm Hg (US: 3 W, 60 s). Scale bar: 50 µm. (T) Fluorescent microscopy images of ROS production of Hela cells cultured with of different samples. (U) Cell viabilities of HeLa cells with different treatments (1: Saline, 2: g‐C_3_N_4_, 3: g‐C_3_N_4_/Pt, 4: g‐C_3_N_4_/Luminol, 5:CPL@M and 6: CPL@M + US). (V) Expression of Caspase‐3 in HeLa cells with different treatments (1: Saline, 2: g‐C_3_N_4_, 3: g‐C_3_N_4_/Pt, 4: g‐C_3_N_4_/Luminol, 5:CPL@M and 6: CPL@M + US). (W) Fluorescence images of CP@M, CPL@M and CPL@M + US penetration in tumor tissue (Blue: g‐C_3_N_4_). Data were expressed as mean ± SE. (*n* = 6). (X) Changes of body weights in various treatment mice during 14 days. (Y) Tumor growth profiles of the mice with different treatments. Reproduced with permission [131]. Copyright 2022, Elsevier B.V.

Cong et al. [131] developed a novel biomimetic nano‐lymphatic vessels (denoted as CPL@M) and investigated their potential applications in reducing tissue interstitial fluid pressure (TIFP) and HT (Figure 5A). CPL@M were fabricated by loading Pt and luminol onto melamine‐derived graphitic carbon nitride (g‐C_3_N_4_), followed by coating with extracted cytomembrane. The obtained CPL@M with Pt nanoparticles uniformly distributed showed a size about 130–150 nm, which was larger than that of g‐C_3_N_4_ (80–100 nm) (Figure 5B–E). As depicted in Figure 5F, luminol underwent a reaction with H_2_O_2_ to emit UV–vis light, which was subsequently absorbed by g‐C_3_N_4_ to emitter blue light. The semiconductor g‐C_3_N_4_/Pt demonstrated superior catalytic activity through a synergistic piezo‐photocatalytic effect for enhancing H_2_O splitting (Figure 5G). Concretely, energy band analysis of CPL@M revealed a VB of 1.98 eV and an *E*_g_ of 2.80 eV, confirming its capability to produce H_2_ from H_2_O under light excitation (Figure 5H,I). As demonstrated by ESR data, CPL@M could generate O_2_^·−^ and ^1^O_2_ under light excitation (Figure 5J,K). The local piezoelectric hysteresis loops of CPL@M illustrated a distinctive butterfly‐shaped pattern characteristic of its robust piezoelectric response (Figure 5L,M). The synergistic coupling effect between light and piezoelectric catalysis in CPL@M was further assessed through ROS levels generated from H_2_O splitting (Figure 5N–P). Besides, in a 1% lactic acid (LA) solution, the coupling piezo‐photocatalytic H_2_O splitting efficiency was quantified at 56.42 µL h^−1^ g^−1^, with H_2_ release efficiency reaching 3134 µmol h^−1^ g^−1^ (Figure 5Q). In cellular studies, the CPL@M group displayed stronger blue fluorescence compared to CPL group due to the enhanced targeting capability of cellular membrane (Figure 5R). As shown in Figure 5S, the CPL@M + US group possessed the most potent anti‐tumor effect with rapid disintegration of 3D cell spheres. These findings confirmed the capacity of CPL@M to penetrate 3D cell spheres and eliminate tumor cells, suggesting potential for reducing TIFP akin to lymphatic drainage simulation, thereby enhancing drug delivery and anti‐tumor efficacy. Intracellular ROS levels were measured in Figure 5T, revealing stronger green fluorescence in the CPL@M group compared to saline, g‐C_3_N_4_/Pt, and g‐C_3_N_4_/Pt@cytomembrane (CP@M) groups, while US enhanced H_2_O splitting, resulting in the highest ROS levels observed in the CPL@M + US group. Accordingly, the CPL@M + US group (group 6) demonstrated the lowest viability at 6.28%, highlighting the enhanced therapeutic effect primarily through piezo‐photocatalysis coupling, including HT facilitated by enhanced catalytic H_2_O splitting and improved Pt‐based chemotherapy by generated ROS (Figure 5U). Figure 5V assessed caspase‐3, a crucial apoptosis marker, showing increased expression correlating with cell death trends. Additionally, the CPL@M + US group displayed enhanced accumulation and penetration in both the tumor center (white box area) and tumor periphery (yellow box area), which affirmed its ability to reduce TIFP by triggering H_2_O splitting, contrasting with the limited penetration of CP@M without light and US excitation (Figure 5W). No significant body‐weight loss was observed, indicating the good biosafety throughout the experiment (Figure 5X). As expected, the CPL@M + US group showed the most remarkable anti‐tumor effects with tumor completely eradicated. This study introduces the novel application of piezo‐photocatalysis in nanolymphatic vessels to reduce TIFP, opening new avenues in nanocatalysis for medicine.

## 3 Photothermal‐Photocatalysis

### 3.1 Principle of Photothermal‐Photocatalysis

Photothermocatalysis is an innovative technology that integrates light into thermocatalytic systems and heat into photocatalytic systems to facilitate co‐catalysis [132, 133, 134]. In this section, we specifically focus on photothermal‐photocatalysis for various cancer treatments. Temperature has been shown to be a critical factor influencing photocatalytic efficiency. The photothermal conversion effect refers to the ability of photothermal materials to convert absorbed solar energy into thermal energy through a series of photophysical processes, subsequently dissipating it into the surrounding environment [135, 136]. The mechanisms underlying photothermal conversion primarily include LSPR, non‐radiative relaxation of semiconductors, and molecular thermal vibration [137, 138, 139]. The enhancement of photocatalytic efficiency through heat manifests in several ways. Firstly, charge separation is improved during the photothermal process. Secondly, this process generates additional energy that promotes the transport of charge carriers in the localized hot zone. Lastly, the increase in local temperature due to the photothermal effect activates adsorbed reactive materials, further enhancing the overall efficiency of the process. Therefore, it is vitally important to make full use of the synergistic effect of photothermal energy and photocatalysis. Besides, the heat from PTT may increase the permeability of cell membranes, making the ROS produced by photocatalysis more effective in penetrating cells and inducing oxidative stress. Conversely, ROS can sensitize tumor cells, making them more susceptible to heat‐induced damage [140, 141, 142]. Some plasmonic semiconductors composed of inexpensive elements address economic concerns while exhibiting narrow *E*_g_ and excellent LSPR‐driven photothermal performance. Notable examples include tungsten oxide (WO_3−_ *_x_*) [143], molybdenum trioxide (MoO_3_) [144] and Cu_2−_ *_x_* S [145], which have been utilized in a wide range of applications, including PTT and photocatalysis. The following sections summarize recent advancements in photothermal‐photocatalysts for various dual‐modal therapies, including PTT/PCT and PTT/PDT, triple‐modal therapies such as PTT/PCT/chemotherapy, PTT/PCT/CDT, PTT/PCT/GT, and PTT/PCT/IT. Additionally, we will explore tetra‐modal therapies that combine PTT/PCT/CDT/chemotherapy, PTT/PCT/CDT/FT, PTT/PCT/FT/IT, and PTT/PCT/GT/IT.

### 3.2 Photothermal‐Photocatalysts for Cancer Therapy

#### 3.2.1 Photothermal‐Photocatalysts for Dual‐Modal Cancer Therapy

Photothermal‐photocatalysts enable the simultaneous application of PTT and PCT using a single agent, significantly enhancing tumor cytotoxicity while minimizing side effects and reducing the risk of resistance. Unlike standalone PTT, which is hindered by heat resistance and inconsistent ablation, or PCT, which faces oxygen dependence and shallow light penetration, the PTT/PCT approach offers a synergistic effect. PTT‐induced heat not only damages cancer cells but also improves tumor oxygenation, boosting ROS generation during PCT, which in turn makes cancer cells more susceptible to heat [10]. This dual‐action strategy ensures more effective and comprehensive tumor destruction, particularly for deep‐seated or resistant cancers.

Two‐dimensional (2D) nanomaterials have garnered significant attention over the past few decades due to their ultrathin structures and remarkable physicochemical properties [146, 147]. Recent research on transition metal dichalcogenides (TMDCs) has intensified, with titanium diselenide (TiSe_2_), a 2D metallic TMDC, emerging as a focal point. TiSe_2_ is particularly noteworthy for its excellent thermoelectric properties and potential applications in PTT and PCT. Currently, TiSe_2_ is known only in its 1T polymorph, exhibiting a formal valence of 3d° for Ti. Bulk single crystals of TiSe_2_ consist of Ti atoms arranged in octahedra coordinated by six Se atoms. These crystals undergo a phase transition around 200 K, transitioning into a charge density wave state characterized by a three‐dimensional commensurate wave vector. This phase transition alters the *E*_g_, which can be further modified through the introduction of transition metals, alkali metals, or organic compounds, leading to varied electronic properties suitable for diverse applications. Furthermore, the elements in TiSe_2_ play crucial roles in various biological processes under physiological conditions, including the synthesis of selenomethionine, selenoxide, and l‐seryl tRNA [148, 149, 150, 151].

**FIGURE 6:**
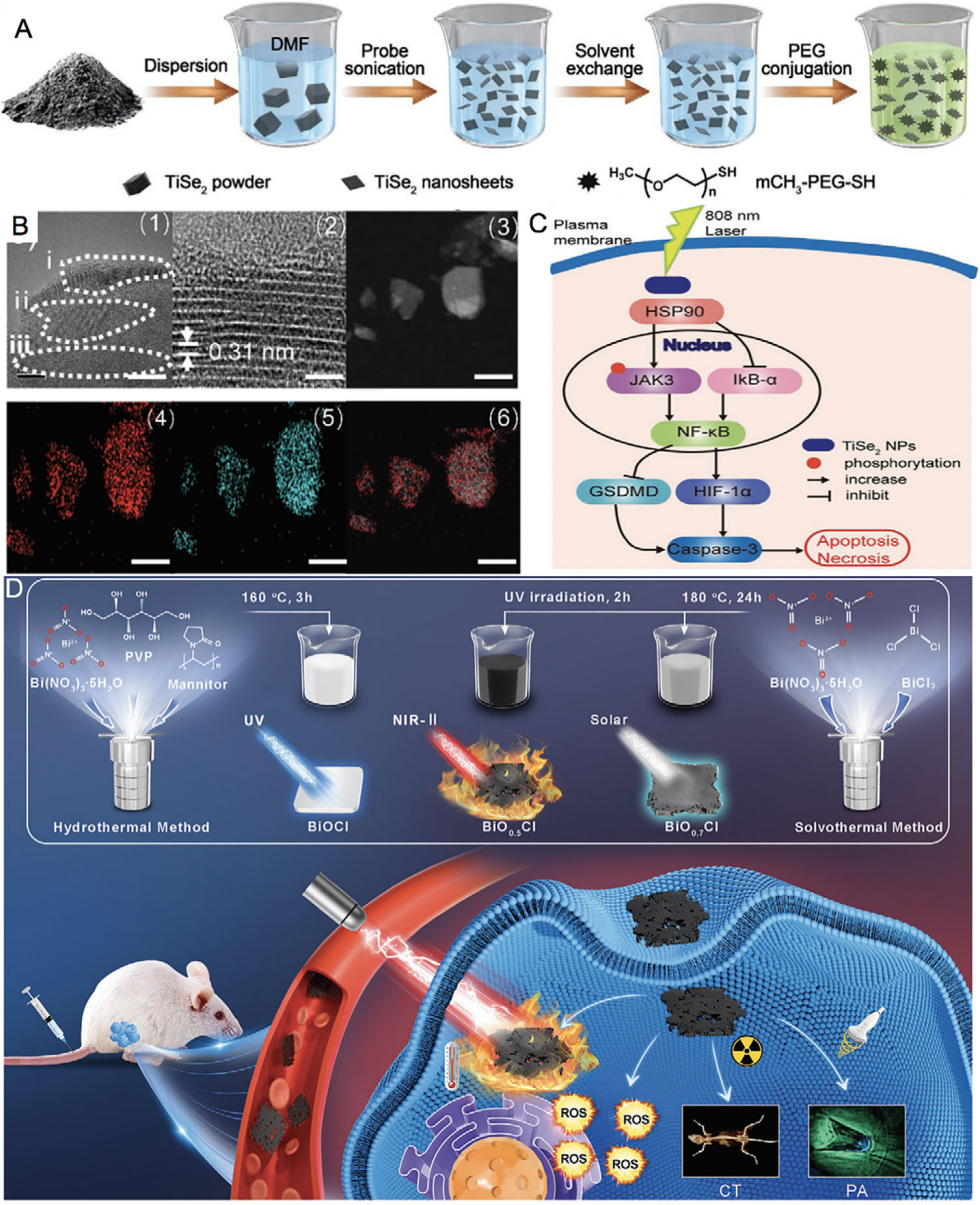
(A) Schematic showing the synthesis of the TiSe_2_ NSs. (B) Microstructure and STEM‐EDS maps of TiSe_2_ NSs ((1) Microstructure of TiSe_2_ NSs, i: dislocation, ii: grain boundaries, iii: amorphous; (2) lattice fringes of TiSe_2_ NSs; (3)–(6) STEM‐EDS maps of TiSe_2_ NSs); scale bar: 10 nm. (C) Schematic of the involved molecular pathway. Reproduced with permission [152]. Copyright 2021, Wiley‐VCH GmbH. (D) Schematic diagram of the preparation of BiO*_x_* Cl NSs with different OV contents and their application for CT/PA dual imaging‐guided NIR‐II synergistic phototherapeutic. Reproduced with permission [156]. Copyright 2024, Wiley‐VCH GmbH.

Duo et al. [152] presented a method for synthesizing two‐dimensional TiSe_2_ nanosheets (NSs) with a sheet‐like morphology using liquid exfoliation. The *E*_g_ of bulk TiSe_2_ is 0.1 eV, while UV spectroscopy and calculations using the Tauc plot method revealed that the *E*_g_ of the synthesized TiSe_2_ NSs was 1.77 eV. This significant change, influenced by various treatments, enhanced both the thermoelectric properties and absorption across the UV to NIR spectrum. Under 808 nm laser irradiation, the temperature of the TiSe_2_ NSs dispersion increased markedly even at a low concentration of 10 ppm, which also demonstrated a positive correlation with power density. Furthermore, the PCE of TiSe_2_ NSs was measured at approximately 65.58%, significantly surpassing that of commercial gold nanorods (GNRs) and indium(II) selenide (InSe). During the process, electrons in TiSe_2_ were excited from the VB to the CB, generating holes. The electrons in the CB reacted with dissolved oxygen molecules to form O_2_^·−^, which oxidized substrates, while the holes in the VB directly oxidized the substrates. Consequently, the separation and recombination of photo‐generated electron–hole pairs improved the photocatalytic activity of TiSe_2_. Using specific fluorescence probes, it was demonstrated that TiSe_2_ NSs effectively generated ·OH and O_2_^·−^ under 808 nm laser irradiation, with the levels of ROS correlating with the duration of irradiation. Importantly, elevated temperatures could enhance catalytic performance and further increase oxidative stress and GSH depletion in cancer cells. Molecular mechanism studies revealed that the combined photothermal and photocatalytic effects induced both apoptosis and necrosis in cancer cells through the heat shock protein90 (HSP90)/JAK3/NF‐κB/IKB‐α/Caspase‐3 pathway. Systematic analysis further indicated that TiSe_2_ NSs degraded significantly and passively accumulated in tumor tissue, promoting their photothermal and photocatalytic effects while exhibiting minimal toxicity. This study underscores the considerable potential of biodegradable TiSe_2_ NSs for effective synergistic phototherapy in cancer treatment (Figure 6A–C).

The introduction of oxygen‐related defects is a promising strategy for significantly enhancing the performance of photocatalysts in cancer therapies. Here are some key advantages: (1) oxygen‐related defects can modify the electronic structure of photocatalysts, broadening light absorption and improving photothermal and photochemical efficiency; (2) defects create localized states that enhance charge carrier mobility, boosting reactive species generation for PCT applications; (3) oxygen defects allow precise adjustment of the *E*_g_, optimizing photocatalytic activity for specific therapeutic needs; (4) oxygen‐related defects can improve the structural stability of photocatalysts, extending their operational lifespan and reducing the frequency of replacements or regenerations [153, 154, 155].

Fang et al. [156] designed a series of bismuth oxychloride (BiO*_x_* Cl) NSs with varying concentrations of oxygen vacancy defects to investigate their impact on the photoelectronic properties. Both experimental and theoretical calculations demonstrated that the *E*_g_ of BiO*_x_* Cl NSs decreased from 3.0 to 0.5 eV as the oxygen vacancy concentration increased to 50%. This reduction in *E*_g_ resulted in a redshift of absorption from the visible/NIR‐I range to the NIR‐II range and even longer wavelengths. The deep‐level oxygen vacancy defects could directly excite local electrons into the CB of BiO*_x_* Cl NSs, significantly lowering the *E*_g_ and enhancing the full‐spectrum absorption capabilities of the BiO*_x_* Cl NSs. Moreover, when photogenerated carriers were efficiently driven by the enhanced internal electric field to generate more ROS, excess carriers underwent nonradiative recombination. This process imparted significant photothermal performance to BiOxCl NSs under NIR‐II laser irradiation. This advancement in carrier utilization demonstrated powerful performance in both PDT and PTT within a single irradiation session. Furthermore, due to its high atomic number, Bi in BiO*_x_* Cl served as an effective contrast agent, exhibiting enhanced CT performance in contrast to that of the clinically used contrast agent ISOVUE (Iopamidol Injection). By engineering oxygen vacancy defects, BiO_0.5_Cl NSs could acquire photoacoustic capabilities, facilitating CT/PA dual‐modal imaging‐guided cancer therapy. PEG modification endowed BiO_0.5_Cl NSs with excellent biosafety and biocompatibility, the unique layered structure of which allowed for effective adhesion to cell membranes and facilitated endocytosis due to their tendency to reduce surface energy. Ultimately, the BiO_0.5_Cl@PEG NSs effectively eliminated tumors in mice when treated with NIR‐II laser irradiation. This research highlights the optimization and potential of wide‐band‐gap and indirect‐band‐gap semiconductor materials (Figure 6D).

CDs are nanoscale carbon‐based materials, typically measuring less than 10 nm in size. They are commonly synthesized from carbon‐rich precursors using various methods, including hydrothermal, microwave‐assisted, and laser ablation techniques [157]. Due to their biocompatibility, photostability, efficient photothermal conversion, high surface area, enhanced charge separation, versatile functionalization, low cost, and simple synthesis, CDs hold significant potential in biomedical applications [158, 159]. It is worth noting that the introduction of oxygen‐related defects into CDs can enhance their performance in PTT and PCT through multiple pathways such as enhanced charge carrier dynamics, increased active sites, tailored *E*_g_, improved reactivity stability and resilience, facilitation of surface reactions, and so on [160].

**FIGURE 7:**
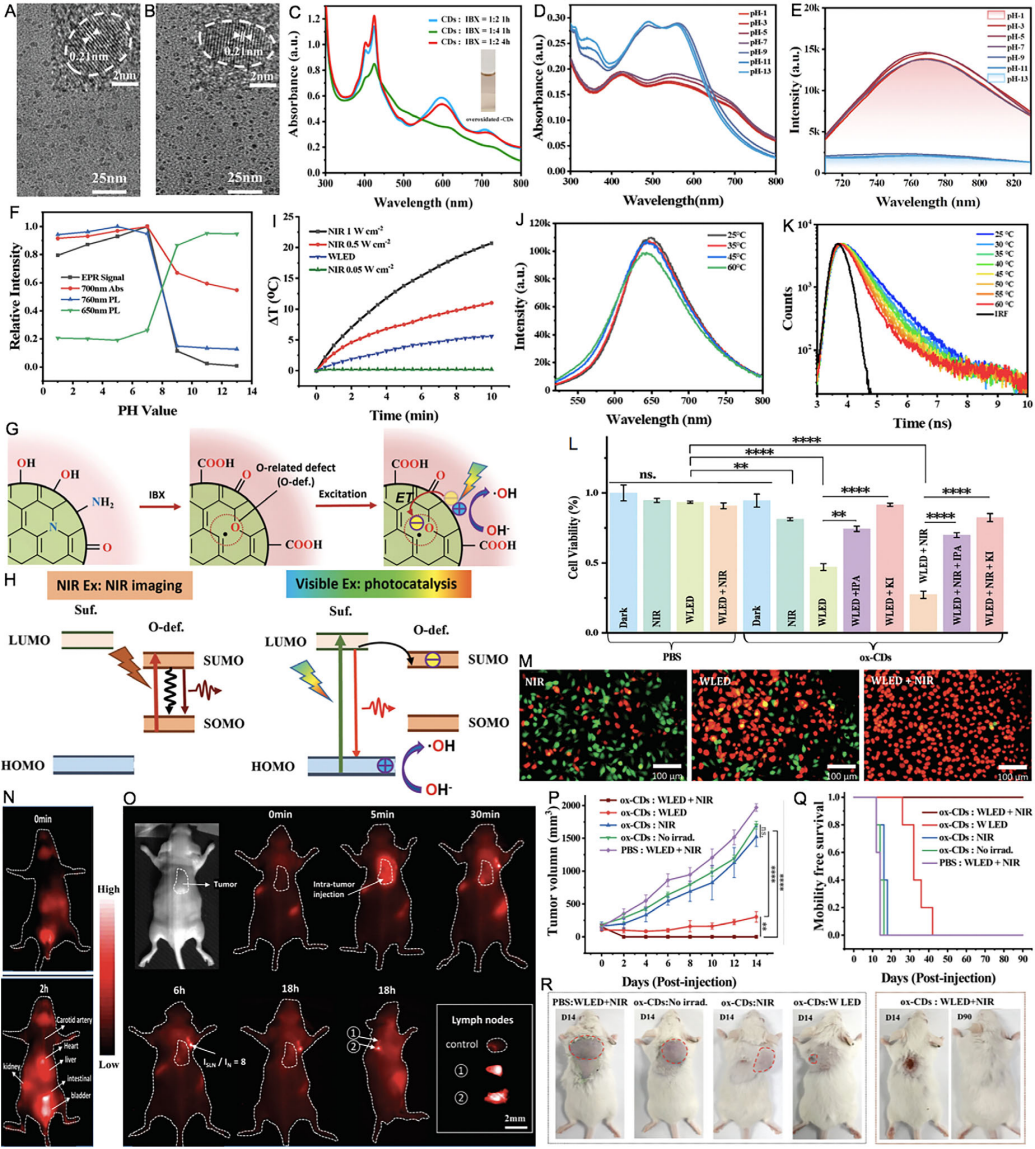
TEM and HRTEM (inset) images of (A) CDs and (B) ox‐CDs. (C) Absorption spectra of CDs after treating with IBX in DMSO for 1 and 4 h with different ratios of CDs and IBX. (D) Absorption spectra of ox‐CD aqueous solutions at different pH. (E) FL spectra of ox‐CD aqueous solutions at different pH under 700 nm excitation. (F) Intensity curves of ESR signals, NIR absorption band at 700 nm, NIR emission peak at 760 nm (700 nm excitation), and red emission peak at 650 nm (600 nm excitation) from ox‐CD aqueous solutions at different pH. (G) Schematic illustrations of the chemical structural changes from CDs to ox‐CDs by IBX and the charge separation process in ox‐CDs under visible light irradiation. (H) Energy level diagrams of ox‐CDs and the photodynamic processes under NIR (left) and visible light (right) excitation. (I) Temperature evolutions of ox‐CD aqueous solutions (200 µg mL^−1^) under 730 nm laser (0.05, 0.5, and 1 W cm^−2^) and 800 lm white LED torch irradiation. (J) Emission spectra and (K) fluorescence lifetime decay curves monitored at 660 nm of ox‐CD aqueous solutions at different temperatures under 510 nm excitation. (L) Cell viabilities of 4T1 cells treated with PBS and 500 µg mL^−1^ ox‐CDs after irradiation by different light sources (5 min) with or without IPA or KI for 24 h. Data represent mean ± SD (*n* = 3). (M) Live/death cell imaging assays of 4T1 cells treated with 500 µg mL^−1^ ox‐CDs after irradiation by different light sources for 10 min (NIR, 1 W cm^−2^ 730 nm laser; W LED, 800 lumen white LED torch). (N) NIR fluorescence images of mice before and after the tail vein injection of ox‐CD aqueous solution (100 µL, 500 µg mL^−1^). (O) NIR fluorescence images of a mouse before and after the intratumoral injection of ox‐CDs aqueous solution (100 µL, 500 µg mL^−1^) at different time points. Bottom right inset: Comparison of the in vitro fluorescence intensity of distant LN (control) and SLNs near the tumor (Ex, 690 nm laser (50 mW cm^−2^); Em, 780 nm long‐pass optical filter/200 ms). (P) Tumor growth curves of 4T1 tumors and representative tumors of mice after different treatments (*n* = 5 for each group), calculated based on the mean tumor size (mean ± SD) for each mouse. (Q) Survival rates of G1–G5 mice. (R) Photographs of G1–G5 mice on day 14 and G5 mice on day 90. Reproduced with permission [161]. Copyright 2023, Wiley‐VCH GmbH.

For instance, Zhang et al. [161] developed a straightforward method to introduce oxygen‐related defects into CDs via post‐oxidation with 2‐iodoxybenzoic acid (IBX) for enhanced noninvasive NIR fluorescent imaging and phototherapy. As shown in Figure 7A, IBX oxidation had minimal impact on the sample sizes. Low IBX concentrations or short reaction times resulted in negligible NIR absorption, whereas excessive oxidation disrupted the conjugated structure of CDs (Figure 7B,C). The absence of ESR signals in alkaline solutions and NIR absorption and emission at 700 and 780 nm suggested that NIR emission originates from oxygen‐related defects with unpaired electrons (Figure 7D–F). The mechanism of NIR emission and photocatalytic processes in ox‐CDs were depicted in Figure 7G,H. IBX oxidation replaced some surface graphitic nitrogen atoms with oxygen, creating unpaired electrons in adjacent carbon atoms and forming NIR emission centers. Under 730 nm laser irradiation, these defects emitted NIR light and heat through radiative and non‐radiative transitions, respectively, without reacting with H_2_O or OH^−^. White LED torch irradiation excited electrons in the surface‐related state, which were trapped by oxygen‐related defects, leading to charge separation and generating photo‐induced holes that oxidized OH^−^ to produce ·OH. The photothermal performance of ox‐CDs was evidenced by a 20.7°C increase in the temperature after laser (1 W cm^−2^) irradiation for 10 min, corresponding to a PCE of 20.4% (Figure 7I). The photoluminescence (PL) lifetime of the red emission decreased with rising temperature, indicating greater energy dissipation from the red emission center at higher temperatures. Consequently, the NIR laser‐induced photothermal effect facilitated electron transfer from the surface‐related state to oxygen‐related defects in the ox‐CDs under visible light irradiation, thereby enhancing the generation of ·OH (Figure 7J,K). As shown in Figure 7L,M, the sharp decrease in cell viability (27%) indicated that the photothermal effect of the ox‐CDs significantly enhanced their white light‐induced cell‐killing ability, as confirmed by the live/dead cell imaging assay. Following intravenous injection of the ox‐CD solution, clear imaging of the heart, carotid artery, kidney, and bladder was achieved within 2 h. Intratumoral injection into a 4T1 tumor‐bearing mouse enabled high‐contrast NIR FLI of sentinel lymph nodes (SLNs) around the tumor within 30 min (Figure 7N,O). The NIR fluorescence signal ratio between SLNs near the tumor and surrounding tissues reached up to 8 and lasted over 18 h, providing extended observation and enhanced contrast for tumor‐associated SLN identification. In vivo therapeutic experiments showed that tumors treated with white LED torch and 730 nm laser irradiation (Group 5) were completely eradicated with no recurrence observed after 90 days (Figure 7P–R). This study represents the first use of the Janus optical properties of pure CDs for noninvasive NIR fluorescent imaging and effective PCT.

#### 3.2.2 Photothermal‐Photocatalysts for Triple‐Modal Therapy

Tumors are characterized by their considerable diversity, complexity, and heterogeneity, which complicate effective treatment, even with the use of dual‐modal therapies. In response to these challenges, researchers are actively working to integrate multiple therapeutic approaches into a single nanoplatform, employing strategic design methods. This integrative strategy aims to enhance anticancer efficacy through synergistic effects while addressing the limitations inherent to individual treatment modalities [162, 163]. The primary methods for enhancing PCT performance have been discussed in the preceding paragraphs. In the following sections, we will focus on the collaboration of various treatment modalities. Specifically, this section reviews several triple‐modal therapies, such as PTT/PCT/chemotherapy, PTT/PCT/CDT, PTT/PCT/GT, and PTT/PCT/IT.

##### 3.2.2.1 PTT/PCT/Chemotherapy

Chemotherapy is a cornerstone of clinical cancer treatment, prompting researchers to investigate a wide range of small‐molecule drugs over recent decades. Despite significant progress in improving its effectiveness and reducing side effects through advanced drug delivery systems, the rise of multidrug resistance (MDR) remains a formidable obstacle [164, 165, 166, 167]. Both PTT and PCT play crucial roles in enhancing the sensitivity of cancer cells to chemotherapeutic agents, thereby circumventing resistance mechanisms. Besides, PTT elevates local temperatures, further increasing the efficacy of both PCT and chemotherapy [168, 169, 170]. The combined effects of these three approaches can lead to more effective tumor destruction, while simultaneously reducing systemic toxicity and minimizing adverse effects. Collectively, these advantages highlight the promise of integrating PTT, PCT, and chemotherapy as a strategic approach in the ongoing battle against cancer.

Yang et al. [171] reported a similar heterostructured photocatalyst, bismuth selenide (Bi_2_Se_3_)/Au, created by in situ depositing Au nanoparticles onto the surface of hollow mesoporous Bi_2_Se_3_. Due to the lower work function of Au nanoparticles, photo‐induced electrons more readily transferred and accumulated on their surfaces, resulting in improved separation of electron–hole pairs and enhanced ROS generation. Furthermore, the Bi_2_Se_3_/Au heterostructures exhibited increased photothermal efficiency, attributed to effective orbital overlap that facilitated accelerated electron migration, as supported by density functional theory calculations. To enhance solubility and biocompatibility, the polymer poly(lactide‐*co*‐glycolide)‐poly(ethylene glycol) (PLGA‐PEG) was applied to the Bi_2_Se_3_/Au structure. Doxorubicin (DOX) was subsequently loaded to create the final formulation, Bi_2_Se_3_/Au@PLGA‐PEG‐DOX (denoted as BS/Au@PP‐DOX), enabling photothermal‐triggered drug release within the system. Additionally, CT with infrared thermal dual‐modal imaging provided immediate diagnostic capabilities, owing to the significant X‐ray attenuation of Au and its strong photothermal performance.

##### 3.2.2.2 PTT/PCT/CDT

CDT represents a novel approach in cancer treatment, utilizing chemical reactions to achieve cytotoxic effects. Unlike conventional therapies such as chemotherapy, radiotherapy, or PDT, which depend on the direct interaction of drugs, radiation, or light with cellular targets, CDT operates through the generation of ROS via chemical processes. This technique often involves transition metal ions, including Fe^2+^, Cu^+^, or Mn^2+^, which catalyze H_2_O_2_ to produce highly reactive ·OH through Fenton or Fenton‐like reactions [172, 173, 174, 175]. Tumors frequently exhibit elevated H_2_O_2_ levels due to heightened metabolic activity and impaired redox regulation. By introducing these catalytic metal ions into the TME, CDT effectively exploits the surplus H_2_O_2_ to selectively generate ROS, specifically targeting cancer cells while preserving healthy tissue. The synergies of thermal, catalytic, and chemical mechanisms can amplify oxidative stress in tumor cells, thereby increasing their vulnerability to treatment [176, 177, 178, 179]. Furthermore, the combination of CDT with PTT and PCT enhances targeting accuracy and minimizes collateral damage to surrounding healthy cells, which helps to reduce systemic side effects. This adaptable multi‐modal approach can be tailored to different tumor types and stages, making it an attractive option for personalized therapy.

**FIGURE 8:**
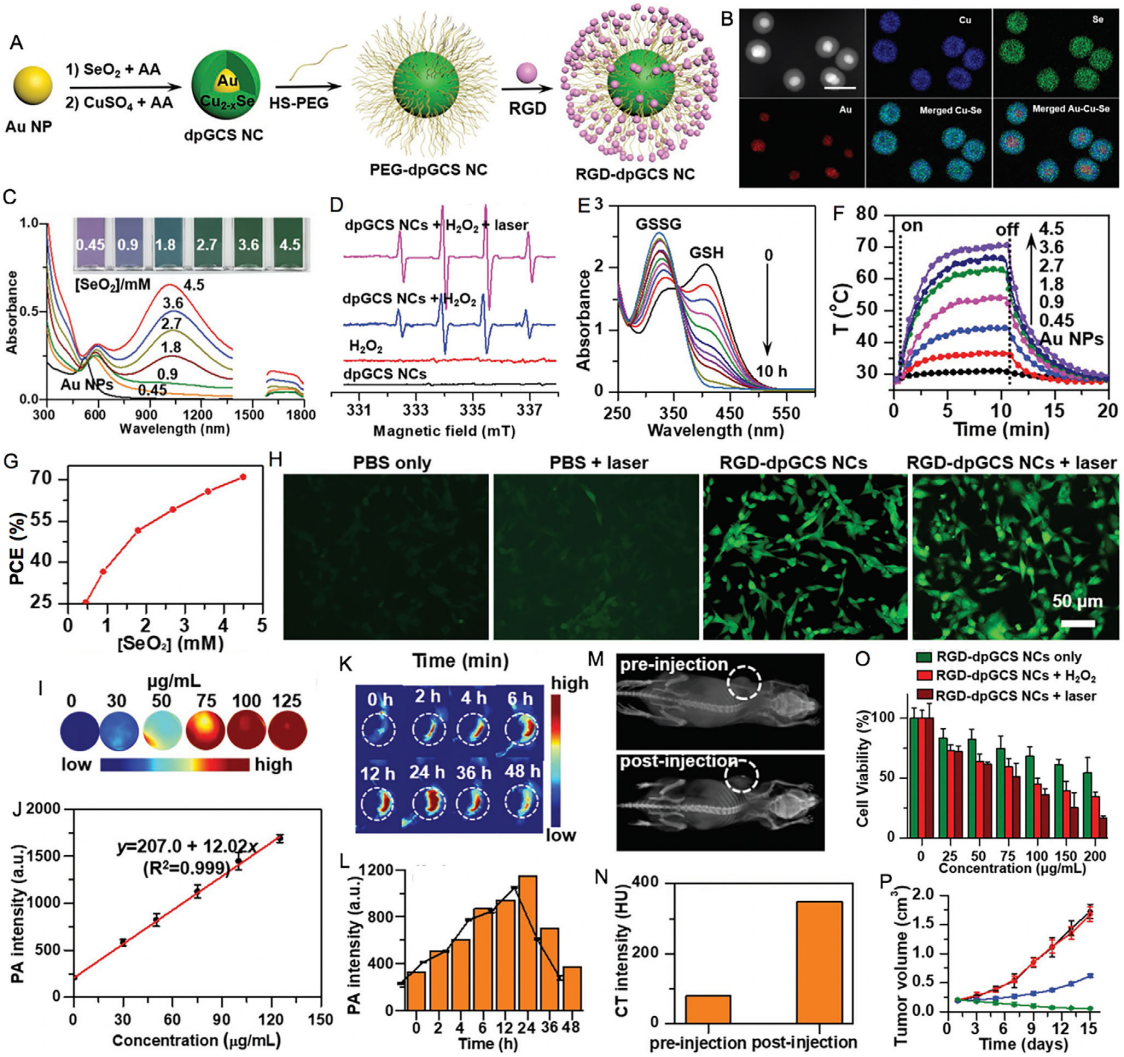
Schematic illustration of (A) the synthesis of NIR‐II plasmonic RGD‐dpGCS NCs. (B) STEM image and elemental distributions of Au, Cu, and Se in dpGCS NCs synthesized with 4.5 mm SeO_2_. Scale bar: 50 nm. The merged images in (B) labeled with Cu─Se and Au─Cu─Se are the overlapped images of the Cu and Se element maps and the Au, Cu, and Se elemental maps, respectively. (C) Optical absorption spectra of aqueous suspensions of dpGCS NCs synthesized with varied SeO_2_ concentrations. The inset in (C) is the corresponding optical photograph. (D) ESR spectra under various conditions with DTNB as a ·OH trapping agent. (E) GSH depletion in the presence of dpGCS NCs plus H_2_O_2_. (F) Temperature profile of aqueous suspensions of dpGCS NCs (45 µg mL^−1^, Au basis) synthesized at various SeO_2_ concentrations. (G) PCE of dpGCS NCs versus SeO_2_ concentration. (H) Intracellular ·OH generation in U87‐MG cells after various treatments, followed by staining with DCFH‐DA fluorescence probe as a ·OH indicator. The dpGCS NCs synthesized with 4.5 mm SeO_2_ were used here. (I) In vitro NIR‐II photoacoustic images of aqueous solutions of dpGCS NCs at various concentrations. (J) The corresponding NIR‐II photoacoustic intensity‐concentration curve. (K) In vivo NIR‐II photoacoustic images of U87‐MG tumor‐bearing mice at different time points postinjection. (L) The corresponding NIR‐II photoacoustic intensity change with the post‐injection time. (M) In vivo CT images. (N) CT intensity of U87‐MG tumor‐bearing mice before and after intratumoral injection of dpGCS NCs (100 µL, 4 mg mL^−1^) for 2 h. (O) Cell viability of U87‐MG cancer cells after various treatments. (P) Changes of tumor volume of U87‐MG tumor‐bearing mice after various treatments. Reproduced with permission [180]. Copyright 2022, Wiley‐VCH GmbH.

Shan et al. [180] introduced an innovative phototheranostic platform based on NIR‐II dual‐plasmonic Au@Cu_2−_ *_x_* Se core–shell nanocrystals (dpGCS NCs). As shown in Figure 8A, the dpGCS NCs were synthesized via a two‐step, selenium (Se) template‐mediated method. The copper selenide (Cu_2−_ *_x_* Se) shell thickness was precisely controlled by adjusting the selenium dioxide (SeO_2_) feed, achieving 38.9 ± 3.4 nm at 4.5 mm (Figure 8B). Elemental mapping confirmed that Au primarily constituted the core, while Cu and Se formed the outer shell. Optical absorption spectra displayed two characteristic bands at approximately 581 and 1030 nm, corresponding to the LSPR of the Au and Cu_2−_ *_x_* Se domains, respectively. Compared to the visible LSPR at 581 nm, the NIR‐II LSPR at around 1030 nm exhibited a greater enhancement with increased SeO_2_ concentration (Figure 8C). In Figure 8D, no significant ESR signals for ·OH were detected with either dpGCS NCs or H_2_O_2_ alone. However, dpGCS NCs + H_2_O_2_ produced an ESR spectrum characterized by a 1:2:2:1 quartet signal, indicative of the DMPO/·OH adduct, revealing a Fenton‐like reaction. Notably, the ESR signal intensity increased significantly under 1064 nm laser exposure (1 W cm^−2^), suggesting enhanced ·OH generation owing to the photoexcitation of plasmonic holes. Due to the existence of Cu^2+^ and H_2_O_2_, the GSH was effectively depleted (Figure 8E). As the SeO_2_ concentration increased from 0.45 to 4.5 mm, the steady‐state temperature of the dpGCS NCs suspension rose sharply from 36.5 to 70.3°C, while Au nanoparticles showed only a modest increase of 2.6°C under the same conditions. This led to a significant enhancement in the PCE of dpGCS NCs, which surged from 25.6% to 71.0%, underscoring the improved performance due to Cu_2−_ *_x_* Se loading (Figure 8F,G). In vitro and in vivo experiments with RGD‐modified dpGCS NCs revealed notable results. As illustrated in Figure 8H, cells treated with PBS alone or PBS plus NIR‐II laser exhibited negligible fluorescence. In contrast, U87‐MG cells treated with RGD‐dpGCS NCs + laser demonstrated significantly stronger green fluorescence than the RGD‐dpGCS NCs group. This fluorescence enhancement resulted from the synergistic effects of combined CDT and PCT, driven by Fenton‐like and photocatalytic reactions alongside GSH depletion. The RGD‐dpGCS NCs showed excellent PAI performance, with signal intensity rising initially and then declining after 24 h post‐injection (Figure 8I–K). The high atomic number of Au also resulted in a marked increase in CT intensity at the tumor site, reaching a HU value of 348 at 2 h post‐injection, up from 80 HU before injection (Figure 8M,N). These findings highlighted the dual‐modal imaging capabilities of RGD‐dpGCS NCs for effective tumor detection. In terms of treatment, RGD‐dpGCS NCs exhibited dose‐dependent cytotoxicity against the U87‐MG cancer cell line, even without H_2_O_2_. The addition of H_2_O_2_ or NIR‐II laser irradiation further enhanced this effect, reducing cell viability to below 20% at 200 µg mL^−1^ of RGD‐dpGCS NCs. This selective toxicity was primarily due to higher levels of H_2_O_2_ in cancer cells, which increased ·OH production through a photo‐enhanced Fenton‐like reaction (Figure 8O). Importantly, the RGD‐dpGCS NCs + laser treatment caused complete tumor growth inhibition, demonstrating superior therapeutic effectiveness of synegistic PTT/PCT/CDT (Figure 8P).

**FIGURE 9:**
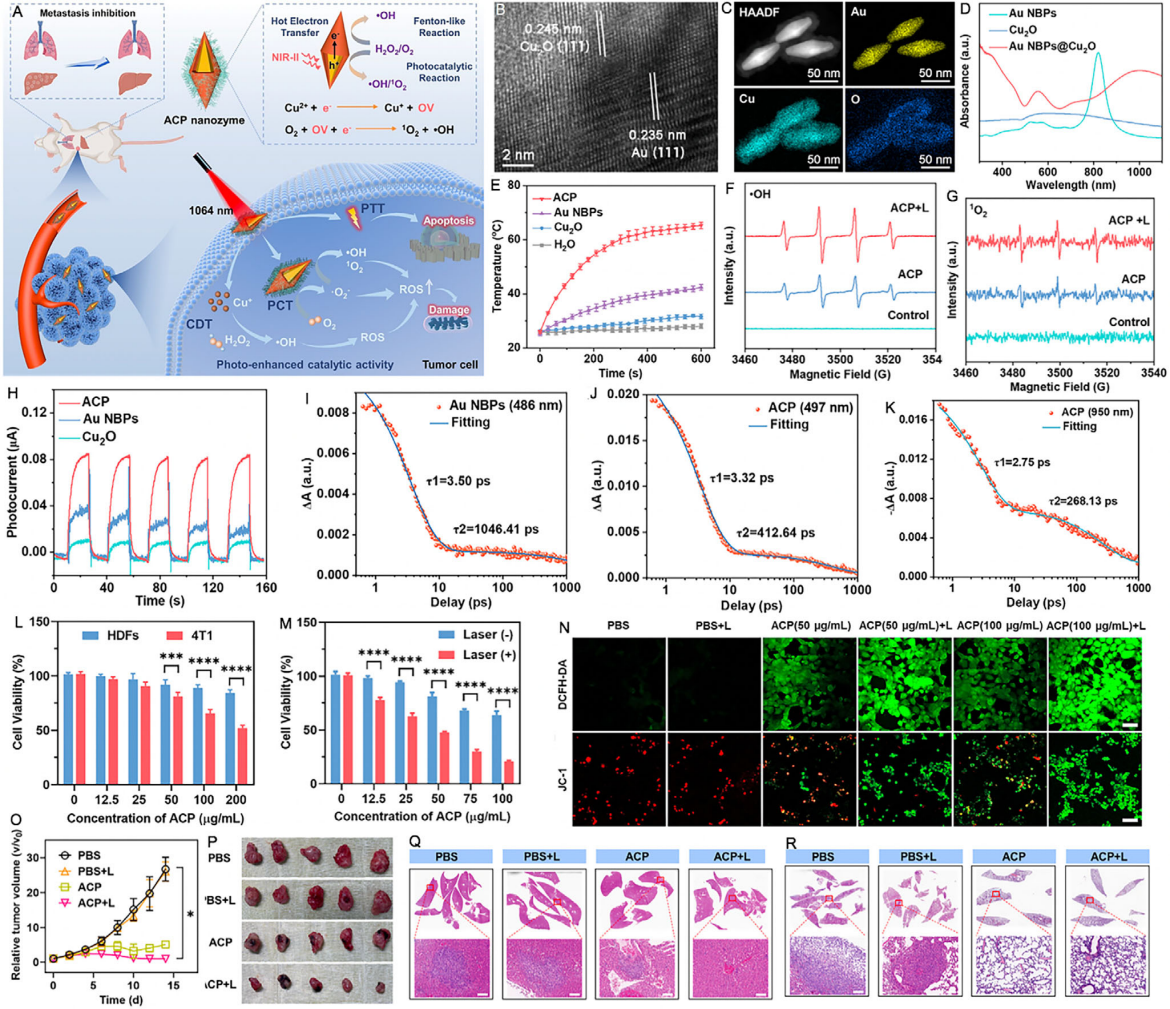
(A) Schematic diagram for the photo‐enhanced catalytic activity mechanism and multifunctional synergistic anti‐tumor mechanism for ACP nanozyme. (B) HR‐TEM image of Au NBP@Cu_2_O nanoparticles. (C) Elemental mapping of Au NBP@Cu_2_O nanoparticles. (D) UV–vis–NIR spectra of Au NBP, Cu_2_O and Au NBP@Cu_2_O nanoparticles. (E) Temperature elevation curves of different samples (100 µg mL^−1^) under 1064 nm laser irradiation (0.75 W cm^−2^). (F) ESR spectra of DMPO/·OH. (G) ESR spectra of TEMP/^1^O_2_. (H) Photocurrent curves of Cu_2_O, Au NBPs and ACP nanoparticles. (I) The kinetics of Au NBPs probed at 486 nm. (J) The kinetics of ACP nanoparticles probed at 497 nm. (K) The kinetics of ACP nanoparticles probed at 950 nm. (L) Relative cell viabilities of HDFs and 4T1 after incubation with ACP. (M) Relative cell viabilities of 4T1 cells after incubation with different concentrations (0, 12.5, 25, 50, 75, 100, and 150 µg mL^−1^) of ACP for 24 h with or without 1064 nm laser irradiation. (N) Intracellular ROS detection by DCFH‐DA in different treatment groups (Scale bars:50 µm) and intracellular mitochondrial membrane potentials by JC‐1 in different treatment groups (Scale bars: 100 µm). The p values were calculated by one‐way ANOVA, ^∗∗∗∗^ *p* < 0.0001, ^∗∗∗^ *p* < 0.001, ^∗∗^ *p* < 0.01, and ^∗^ *p* < 0.05. (O) Changes in the relative tumor volume of mice with different treatments (*n* = 5). (P) Photographs of tumors dissected from each group of mice on the 14th day. (Q, R) ACP inhibited the formation of liver and lung metastases in 4T1 tumor‐bearing mice (scale bar: 100 µm). The *p* values were calculated by one‐way ANOVA, ^∗∗∗∗^ *p* < 0.0001, ^∗∗∗^ *p* < 0.001, ^∗∗^ *p* < 0.01, and ^∗^ *p* < 0.05. Reproduced with permission [181]. Copyright 2023, Elsevier Ltd.

Zhao et al. [181] synthesized a gold nanobipyramid@cuprous oxide (Au NBP@Cu_2_O) nanozyme for effective phototherapy of breast cancer (Figure 9A). High‐resolution (HR)‐TEM analysis revealed lattice spacings of 0.235 and 0.245 nm, corresponding to the (111) planes of cubic Au and Cu_2_O, respectively (Figure 9B). The Cu_2_O coating shifted the LSPR peak of Au NBP from the NIR‐I to NIR‐II region, with the composites (derived from Au NBPs with an aspect ratio of 3.3) showing a strong absorption peak at 1064 nm (Figure 9C,D). Under 1064 nm laser irradiation (0.75 W/cm^2^) for 5 min, the polyvinyl pyrrolidone‐modified Au NBP@Cu_2_O (ACP) temperature rose to 60°C, demonstrating excellent photothermal performance with a PCE of 58% (Figure 9E). In addition to the Cu^+^‐mediated Fenton‐like reaction, the Au NBP@Cu_2_O nanoheterostructure could capture hot electrons generated by equipartition excitations and enhance electron–hole separation when subjected to 1064 nm laser irradiation, thereby facilitating ROS production (Figure 9F,G). As shown in Figure 9H, the unique structure of ACP with significantly enhanced photocurrent effectively promoted the separation of photo‐induced electrons and holes. Femtosecond transient absorption spectroscopy (fs‐TA) was used to analyze photogenerated carrier dynamics (Figure 9I–K). The decay times for Au NBP at 486 nm were 3.50 ps and 1046.41 ps, while for the Au NBP@Cu_2_O heterostructure at 497 nm, they were 3.32 and 412.64 ps. Cu_2_O integration accelerated electron‐phonon coupling, facilitating excitation. Ground state bleaching showed Au NBP lifetimes at 732 nm of *τ*_1_ = 2.96 ps and *τ*_2_ = 44.68 ps, linked to electron trapping. In contrast, the Au NBP@Cu_2_O structure exhibited a longer *τ*_2_ = 268.13 ps at 950 nm, indicating reduced charge recombination. These results suggested that Au NBP integration enhanced carrier excitation and extended recovery time, promoting efficient hot electron injection into Cu_2_O. Due to the higher endogenous H_2_O_2_ levels in tumor cells compared to normal cells, ACP displayed minimal toxicity toward human dermal fibroblasts (HDFs) while demonstrating dose‐dependent cytotoxicity against 4T1 tumor cells (Figure 9L). As shown in Figure 9M, laser irradiation significantly enhanced the tumor cell‐killing effect. At a concentration of 100 µg/mL, ACP nanozymes inhibited nearly 80% of 4T1 cells under NIR‐II laser irradiation, compared to 36% inhibition without laser treatment. Intracellular ROS production was reflected in the increased green fluorescence after ACP treatment for 12 h, which could be further intensified with NIR‐II laser exposure. The ROS‐induced oxidative stress triggerred mitochondrial depolarization, thereby exacerbating apoptosis (Figure 9N). As shown in Figure 9O,P, the ACP + L group exhibited nearly complete tumor growth inhibition, indicating a potential synergistic effect between NIR‐II PTT and enhanced catalytic properties in cancer treatment. In the PBS and PBS + L groups, significant cancer cell infiltration was observed in the liver and lungs, but metastasis was notably reduced in the ACP group. Remarkably, only the ACP + L group preserved the structure of liver and lung tissues (Figure 9Q,R). These findings revealed that the photothermal and catalytic synergistic treatment with ACP effectively inhibited breast cancer metastasis to the liver and lungs. This study provides valuable insights for the design of novel nanocatalytic materials aimed at multifunctional synergistic anti‐tumor therapy.

##### 3.2.2.3 PTT/PDT/GT

GT has emerged as an innovative treatment modality that employs various gasotransmitters to trigger tumor cell death. This approach is distinct from traditional chemotherapy and radiotherapy, as it presents a more environmentally sustainable option with minimal toxicity [182, 183]. Among these gasotransmitters, hydrogen (H_2_) plays a pivotal role in photocatalytic reactions, typically generated through processes like H_2_O splitting [184]. HSPs are commonly linked to cancer cell survival and resistance against treatments involving thermal or oxidative stress. They are integral to key processes associated with malignant transformation and tumor progression, including the promotion of independent growth, evasion of apoptosis and senescence, neoangiogenesis, and the invasion and metastasis of surrounding tissues [185, 186]. Research indicates that HSP activation is contingent upon the binding and hydrolysis of adenosine triphosphate (ATP) at the protein's N‐terminal domain. Notably, H_2_ therapy has shown promise in causing mitochondrial damage in tumor cells, leading to a notable reduction in ATP levels [187, 188]. The triple combination of PTT, PDT, and H_2_ can harnesses the heat generated by PTT to enhance tumor oxygenation, thereby improving PDT efficacy, while also triggering controlled H_2_ release. Simultaneously, H_2_ acts as a selective antioxidant, protecting healthy tissues from oxidative damage, while sensitizing cancer cells to ROS and heat. This creates a powerful yet low‐toxicity anticancer effect.

**FIGURE 10:**
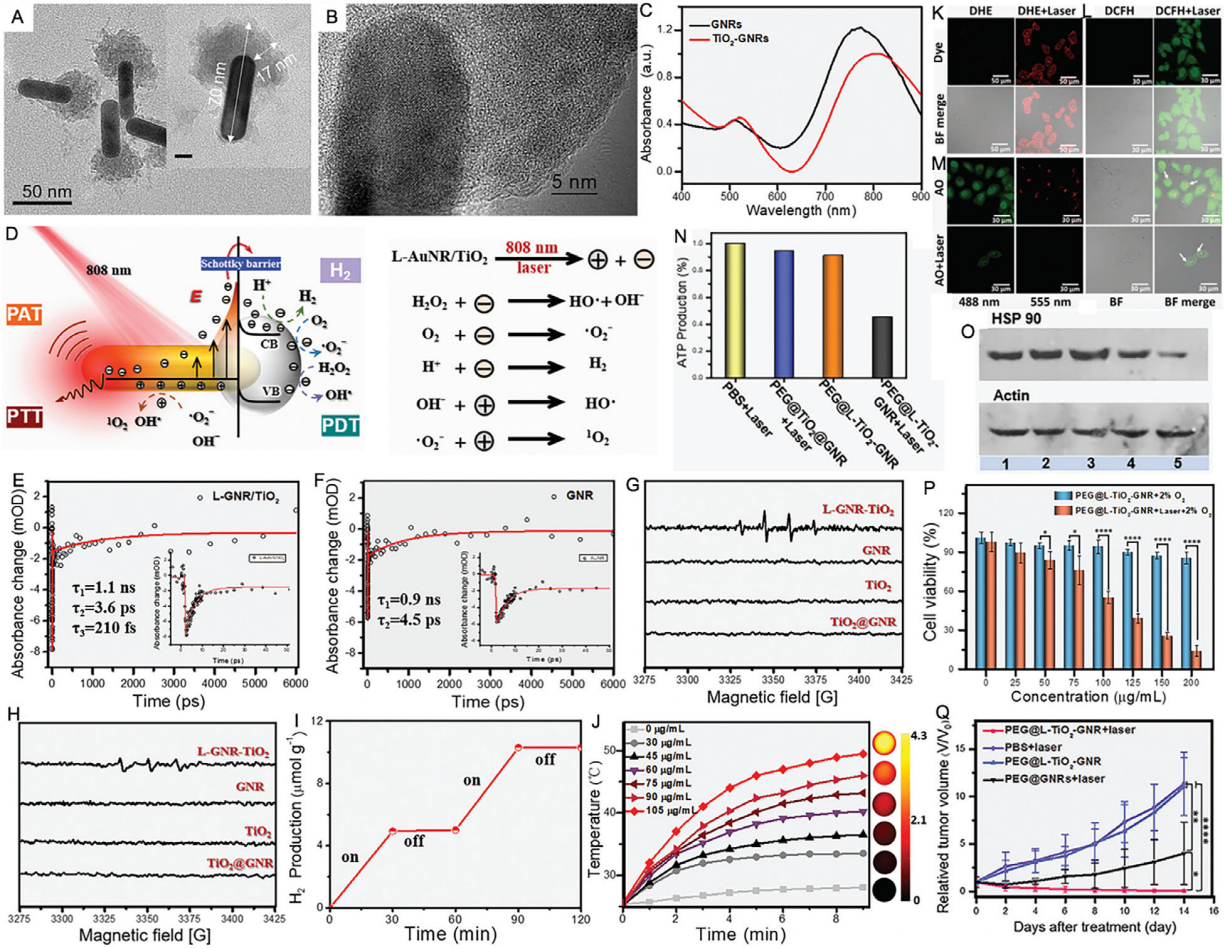
(A) TEM image of L‐TiO_2_‐GNR nanoparticles. (B) HRTEM image of the interface between TiO_2_ and Au. (C) UV–vis absorbance spectra of GNRs and L‐TiO_2_‐GNR nanoparticles. (D) Proposed mechanism for ROS generation and photothermal effects by L‐TiO_2_‐GNR nanoparticles under 808 nm irradiation. Femtosecond time‐resolved transient absorption of (E) L‐TiO_2_‐GNR and (F) GNR (excited at 650 nm). ESR spectra for L‐TiO_2_‐GNR + 1 × 10^−3^ m H_2_O_2_ + laser, GNR + 1 × 10^−3^ m H_2_O_2_ + laser, TiO_2_ + 1 × 10^−3^ m H_2_O_2_ + laser, and TiO_2_@GNR + 1 × 10^−3^ m H_2_O_2_ + laser (laser: 808 nm) in the presence of (G) DMPO and (H) 2,2,6,6‐tetramethyl‐1‐piperidinyloxyl (TEMPO). (I) Responsiveness of L‐TiO_2_‐GNR to near‐infrared photocatalytic H_2_ gas production. (J) Effect of time and concentration on photothermal properties of L‐TiO_2_‐GNR and PAI. (K) Dihydroethidium (DHE) and (L) DCFH‐DA staining for detecting of ROS produced by L‐TiO_2_‐GNR nanoparticles in MCF‐7 cells under 808 nm irradiation. (M) CLSM images of acridine orange (AO) staining for lysosomal integrity. (N) The inhibition rate of ATP in the MCF‐7 cells after receiving different treatments. (O) The changes of HSP90 content in MCF‐7 cells under different conditions (1. Control; 2. PEG@L‐TiO_2_‐GNR; 3. PEG@GNR + Laser; 4. PEG@TiO_2_@GNR + Laser; 5. PEG@L‐TiO_2_‐GNR + Laser). (P) Methylthiazolyldiphenyl–tetrazolium bromide (MTT) of MCF‐7 cells under hypoxic conditions (2% O_2_) treated with PEG@L‐TiO_2_‐GNR nanoparticles followed by irradiation at 808 nm. All the data of figures represent the mean value ± SD (*n* = 4). *P*‐values were calculated using ordinary one‐way ANOVA, *p < 0.05, **p < 0.01, ***p < 0.001, and ****p < 0.0001. (Q) Relative tumor volume (Each value represents the mean ± SD, *n* = 5) of mice after different treatments. *P*‐values were calculated using ordinary one‐way ANOVA, *p < 0.05, **p < 0.01, ***p < 0.001, and ****p < 0.0001. Reproduced with permission [191]. Copyright 2022, Wiley‐VCH GmbH.

Up to now, advanced H_2_ generators have been developed to effectively transport and release H_2_ at specific sites [189, 190]. Ge et al. [191] introduced an innovative asymmetric lollipop nanostructure by integrating GNRs with TiO_2_ to exploit the synergistic effects of PDT, PTT, and H_2_ therapy. The L‐TiO_2_‐GNR nanoparticles, synthesized via wet chemical methods, consist of GNRs (∼12 × 45 nm) with a single TiO_2_ particle (16 ± 2 nm) attached at one terminus (Figure 10A,B). TiO_2_ deposition induced a significant NIR absorption redshift (from 775 to 805 nm) due to localized refractive index changes (Figure 10C). Photophysical characterization revealed that under irradiation, GNR‐generated hot electrons efficiently overcame the TiO_2_ Schottky barrier, with femtosecond transient absorption spectroscopy demonstrating ultrafast electron injection into TiO_2_ within 210 fs (*τ*_3_), followed by sequential energy dissipation through electron‐phonon scattering (*τ*_2_ = 3.6 ps) and charge recombination (*τ*_1_ = 1.1 ns), significantly faster kinetics than observed in bare GNRs (*τ*_1_ = 0.9 ns, *τ*_2_ = 4.5 ps) (Figure 10D–F). This unique electron dynamics enables L‐TiO_2_‐GNR to function as a dual‐mechanism photosensitizer, where both Type I (dominant electron‐transfer pathway) and Type II (energy transfer) photodynamic processes operate concurrently (Figure 10G,H). Beyond its remarkable ROS generation capability, the nanocomposite demonstrated catalytic H_2_ production in the presence of sacrificial agents for GT, while simultaneously exhibiting concentration‐dependent photothermal heating and excellent PAI performance (Figure 10I,J). Cellular studies confirmed efficient laser irradiation‐triggered ROS generation with lysosomal localization, inducing lysosomal damage‐mediated apoptosis (Figure 10K–M). Importantly, the generated H_2_ suppressed cellular stress responses by reducing HSP90 levels through mitochondrial impairment (Figure 10N,O). As demonstrated in Figure 10P, L‐TiO_2_‐GNR with laser treatment exhibited potent cytotoxic effects even under hypoxic conditions (2% O_2_). In vivo evaluation showed complete tumor ablation without recurrence for the PEG@L‐TiO_2_‐GNR (+laser) group, in stark contrast to the temporary regression observed with GNRs alone (4‐fold regrowth) and control groups (11‐fold expansion) (Figure 10Q). Collectively, this work establishes an innovative multimodal therapeutic platform that synergistically combines Type I PDT, mild PTT, and H_2_ therapy to effectively address hypoxic tumors.

##### 3.2.2.4 PTT/PCT/IT

IT represents a promising frontier in biomedicine, attracting considerable research attention for its capacity to leverage the immune system to generate effective systemic antitumor responses [192, 193]. Within the TME, tumor‐associated macrophages (TAMs) and regulatory T (Treg) cells are pivotal in tumor growth and metastasis. TAMs, among the most prevalent non‐neoplastic immune cells in tumors, infiltrate the TME and typically exhibit an M2 phenotype. This phenotype is characterized by the secretion of anti‐inflammatory cytokines that foster immunosuppression and facilitate tumor progression [194, 195]. In contrast, M1 macrophages possess pro‐inflammatory properties that enhance immune responses, producing cytokines that activate cytotoxic CD8^+^ T cells and promote antitumor immunity [196, 197]. Researchers are thus investigating therapeutic approaches to reprogram the TME, aiming to create a pro‐inflammatory environment, enhance CD8^+^ T cell infiltration, and reduce immunosuppressive cells such as M2 macrophages and Tregs [198]. Two significant areas of focus in nanomedicine are immunogenic cell death (ICD) and immune checkpoint blockade (ICB). ICD refers to a form of cell death that initiates an immune response through the release of danger signals. Various cancer therapies including PTT, PDT, chemotherapy, and radiation therapy have been demonstrated to induce ICD [199, 200, 201]. During this process, dying cancer cells release molecules like damage‐associated molecular patterns (DAMPs) and tumor‐associated antigens (TAAs), which activate the immune system to target and eliminate cancer cells, thereby establishing antitumor immune memory [202]. ICB is a cancer IT approach that targets proteins on immune or tumor cells, which act as brakes on the immune response. These immune checkpoints, such as PD‐1 (programmed cell death protein 1) and CTLA‐4 (cytotoxic T‐lymphocyte‐associated protein 4), are found on T cells and typically prevent the immune system from attacking healthy cells. When these checkpoints interact with their ligands (e.g. PD‐L1 on tumor cells), T cell activity is suppressed, allowing tumors to evade immune detection [203, 204].

**FIGURE 11:**
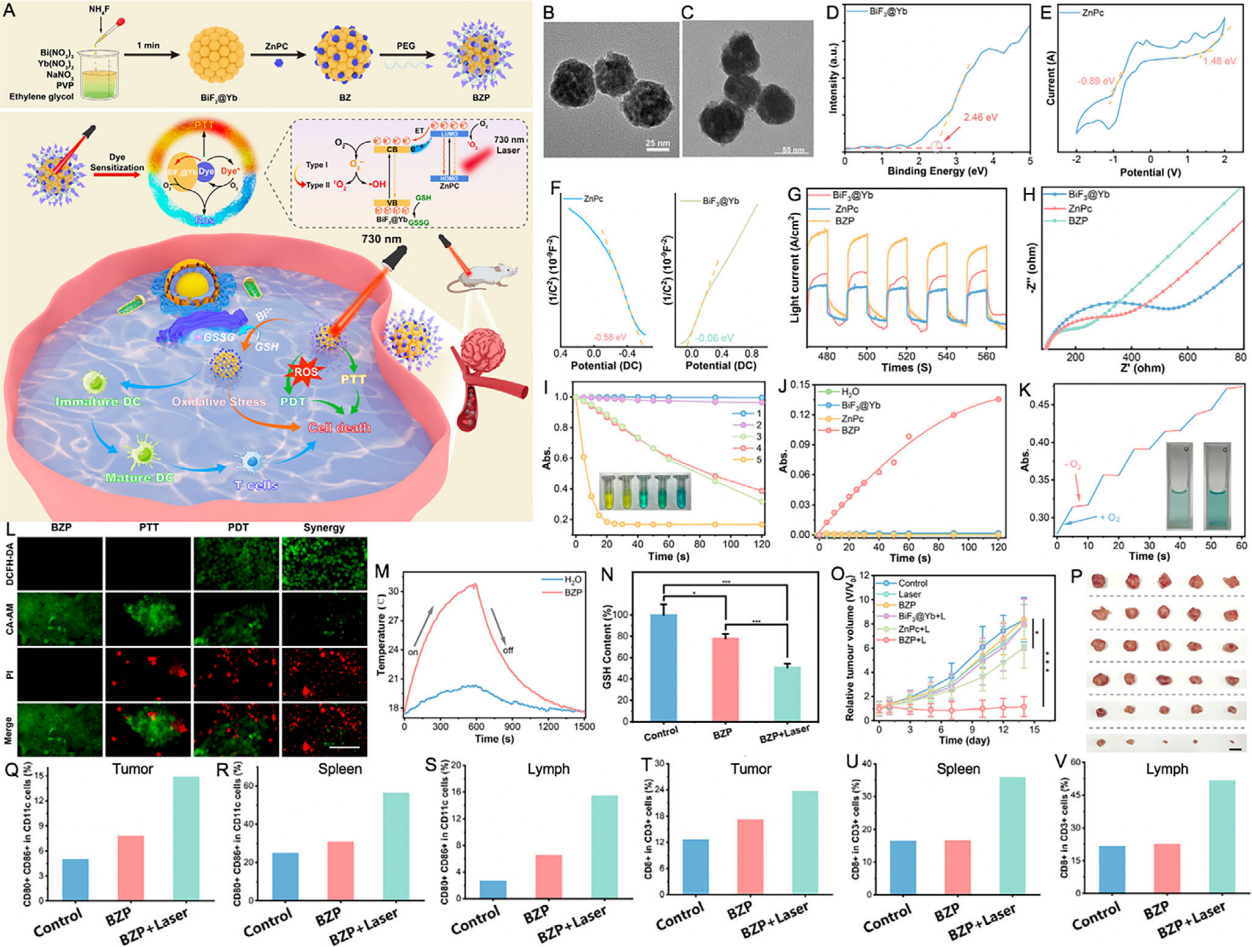
(A) Schematic diagram of BZP synthesis process and its semiconductor nanocatalyst for rare earth doping and dye sensitization enhanced photo‐immune activation therapy of mouse breast tumor. (B) TEM of BiF_3_@Yb. (C) TEM image of BZP. (D) VB spectrum of BiF_3_ @Yb. (E) Cyclic voltammetry pattern of ZnPc. (F) Mott–Schottky curves of ZnPc and BiF_3_@Yb. (G) Time‐dependent photocurrent response and (H) electrochemical impedance spectra of BiF_3_@Yb, ZnPc, and BZP. (I) Normalized absorbance at 421 nm of 1,3‐diphenylisobenzofuran (DPBF) versus time under 730 nm light irradiation for different groups, inset: photos of color change of different groups after the reaction (1. H_2_O; 2. BiF_3_@Yb; 3. BiF_3_@Yb + ZnPc; 4. ZnPc; 5. BZP). (J) Absorbance at 652 nm versus time under 730 nm light irradiation for different groups mixed with tetramethylbenzidine (TMB). (K) Normalized absorbance at 652 nm versus irradiation time trend, inset: color change of the solution before and after the reaction, left: BZP, right: BZP + light (60 s). 730 nm light: 1.0 W cm^−2^. (L) live/dead cell staining in different groups (scale bar: 100 nm). (M) Photothermal heating‐cooling curves of BZP and H_2_O. (N) The changes of relative GSH content (mean ± SD, *n* = 5) in 4T1 tumor cells treated in different groups. (O) Tumor growth kinetics of different groups. (P) Images of excised tumors from different groups after 14‐day treatment (scale bar: 1 cm). (Q–S) Representative data of mature DCs counting in various tissues after treatment with different groups. (T–V) Relative abundance of CD8^+^ and CD4^+^ T cells in various tissues after treatment with different groups. Reproduced with permission [205]. Copyright 2024, Elsevier Ltd.

By incorporating dye molecules onto the semiconductor surface, the absorption spectrum is broadened, allowing for more efficient use of the NIR light. This process also influences the transport pathways of photogenerated carriers, prolonging their lifetime and improving catalytic activity. For instance, He et al. [205] proposed a strategy for optimizing the band structure of Bi‐based semiconductors through rare earth element doping and dye sensitization for synergistic breast cancer treatment (Figure 11A). As shown in Figure 11B,C, ytterbium (Yb)‐doped bismuth fluoride (BiF_3_@Yb) served as a wide‐bandgap semiconductor with a spherical and mesoporous structure, which was loaded with ZnPc and surface‐modified with PEG to obtain BiF_3_@Yb‐ZnPc‐PEG (denoted as BZP). Bandgap analysis revealed that BiF_3_@Yb (15% doping) had a bandgap of 3.33 eV, with a VB at 2.46 eV, corresponding to a CB at −0.87 eV (Figure 11D). ZnPc cyclic voltammetry showed an oxidation peak at −0.89 eV and a reduction peak at 1.48 eV, leading to a HOMO level of 0.68 eV and a LUMO level of −1.69 eV (vs. standard hydrogen electrode) (Figure 11E). The Fermi level of BiF_3_@Yb (−0.06 eV) was higher than that of ZnPc (−0.58 eV), driving photoexcited electrons from ZnPc to BiF_3_@Yb, which facilitated charge separation and prolonged carrier lifetimes (Figure 11F). Photoelectrochemical measurements confirmed this, showing that BZP exhibited significantly higher photocurrent intensity than ZnPc or BiF_3_@Yb alone under intermittent illumination (five 10‐s on/off cycles), indicating enhanced charge transfer efficiency due to dye sensitization (Figure 11G). Electrochemical impedance spectroscopy (EIS) further demonstrated that BZP had the smallest semicircle radius, confirming its superior charge‐transfer efficiency and electron mobility, key factors for photocatalytic performance (Figure 11H). Under 730 nm laser irradiation, ZnPc generated ^1^O_2_ via energy transfer to molecular oxygen. Simultaneously, photoexcited electrons moved from the HOMO to the LUMO of ZnPc and were transferred to the CB of BiF_3_@Yb due to favorable Fermi level alignment. These electrons reacted with oxygen to form O_2_^·−^, which further produced ^1^O_2_ and ·OH (Figure 11I–K). Meanwhile, the holes in the VB of BiF_3_@Yb oxidized GSH, suppressing charge recombination and enhancing ROS production. The photothermal effect induced tumor ablation, improved local blood perfusion, and further promoted ROS generation. Additionally, Bi^3+^ in BiF_3_@Yb coordinated with GSH, increasing oxidative stress and enabling biodegradation and metabolic clearance (Figure 11L–N). In addition to direct tumor destruction, the synergistic effects of ROS and hyperthermia stimulated DC maturation and T cell activation, enhancing antitumor immunity (Figure 11O–V). Such combined strategies of elemental doping and dye sensitization effectively enhanced photocatalytic activity and immune activation, offering a promising platform for semiconductor‐based cancer therapy.

#### 3.2.3 Photothermal‐Photocatalysts for Tetra‐Modal Therapy

Beyond triple‐modal therapy, researchers are exploring tetra‐modal therapy, which integrates four different therapeutic modalities to enhance cancer treatment. This multi‐faceted approach allows for the targeting of various cellular pathways and molecular targets, thereby providing a comprehensive strategy that addresses the inherent heterogeneity of cancer cells in tumors. Additionally, tetra‐modal therapy may facilitate lower dosages of each treatment component, helping to reduce systemic toxicity and minimizing adverse effects on healthy tissues. The synergistic effects of these combined modalities can significantly improve therapeutic outcomes, potentially leading to better long‐term cancer control and increased patient survival rates [206, 207, 208]. In this section, we will introduce several tetra‐modal treatment combinations, such as PTT/PCT/CDT/chemotherapy, PTT/PCT/CDT/FT, PTT/PCT/FT/IT, and PTT/PCT/GT/IT.

##### 3.2.3.1 PTT/PCT/CDT/Chemotherapy

Yan et al. [209] developed ultrathin, porous nitrogen‐doped carbon‐coated stoichiometric CuSe heterostructures (CuSe/NC) using a simple, environmentally friendly one‐pot hydrothermal method. NC is well‐known for enhancing the photocatalytic activity of semiconductors by improving electron–hole pair separation and inhibiting rapid recombination. The porous structure of the ultrathin NC shells retained surface coordination sites (Cu^2+^) from the CuSe cores, facilitating DOX loading. Additionally, DNA was conjugated to the surface of CuSe/NC‐DOX via multiple interactions, enhancing biocompatibility and cellular uptake. This innovative nanoplatform for multimodal cancer therapy offered several key features: (1) controlled release of DOX was achieved through an “AND” logic mechanism that responded to the dual stimuli of low pH and elevated GSH levels in TME, minimizing premature drug leakage and reducing side effects on normal tissues; (2) the release of DOX depleted intracellular GSH levels, leading to increased endogenous H_2_O_2_ concentrations; and (3) under NIR light irradiation, the CuSe/NC generated hyperthermia and O_2_‐independent free radicals, promoting ·OH production via a Fenton‐like reaction, effectively addressing tumor hypoxia and improving therapeutic specificity.

##### 3.2.3.2 PTT/PCT/CDT/FT

Ferroptosis is a regulated form of cell death marked by lipid peroxide accumulation and iron‐dependent oxidative stress. Unlike apoptosis or necrosis, it results from disrupted iron homeostasis and depleted antioxidants, particularly GSH. Given its unique mechanisms, ferroptosis is being explored as a potential therapeutic target in cancer treatment, as inducing ferroptosis in tumor cells could enhance the efficacy of existing therapies [210, 211]. The synergistic application of PTT, PCT, CDT, and FT offers a more thorough approach to cancer cell destruction. While PTT and PCT cause immediate cellular damage, CDT and FT can extend therapeutic effects by initiating prolonged cell death pathways. Furthermore, the damage from these treatments can release tumor antigens, stimulating an immune response and enhancing systemic immunity against cancer [212, 213].

**FIGURE 12:**
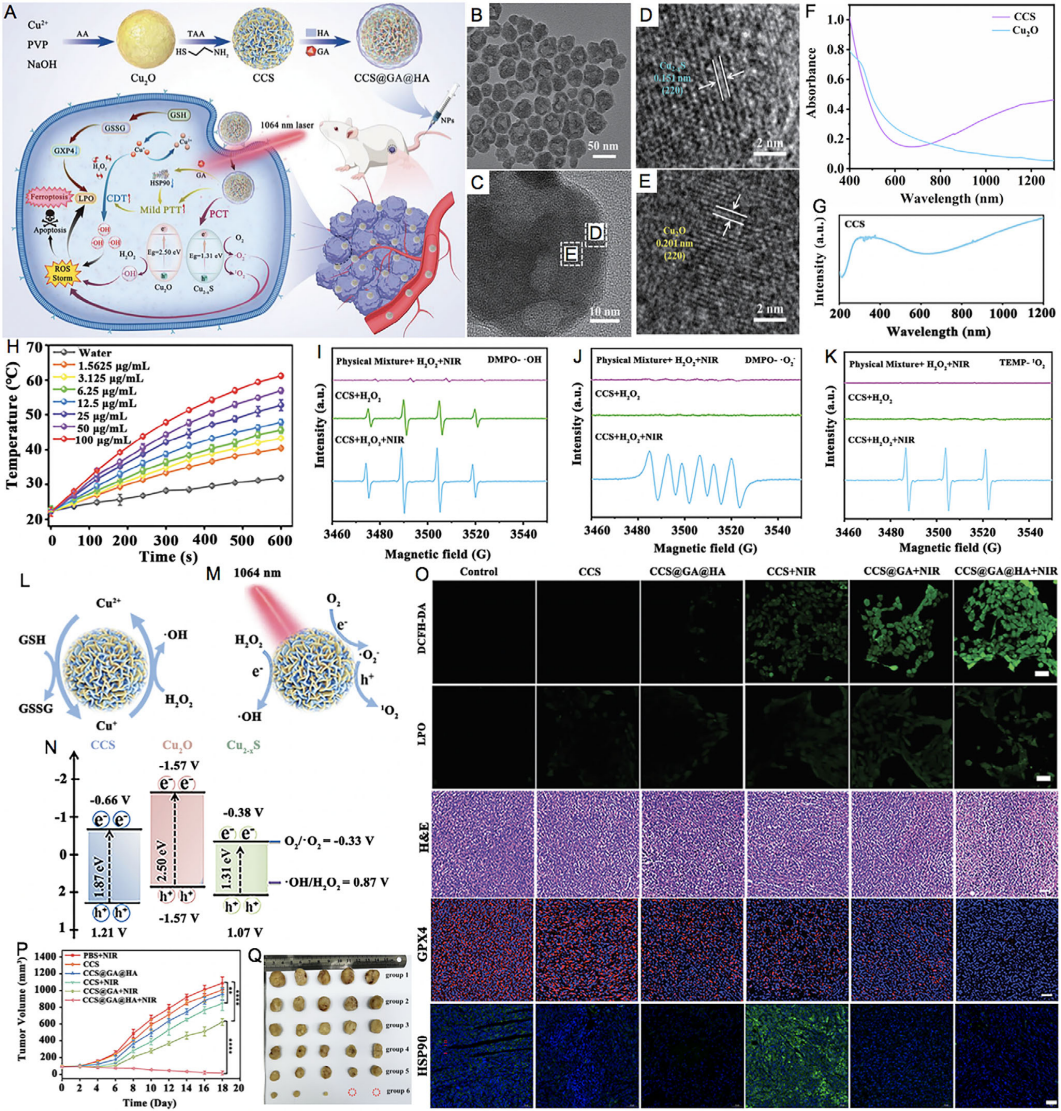
(A) Schematic illustration of the synthetic process of CCS@GA@HA and multi‐modal treatments of CCS@GA@HA nanoplatforms. (B) TEM and (C) HRTEM images of CCS nanoparticles. (D) and (E) were the corresponding images amplified from (C) HRTEM of CCS nanoparticles. (F) UV–vis absorption spectrum of CCS nanoparticles and Cu_2_O in water. (G) UV–vis diffuse reflectance spectra of CCS, Cu_2_O and Cu_2−_ *_x_* S. (H) Temperature elevation curves of CCS nanoparticles aqueous solutions with different concentrations (0–100 µg mL^−1^, 1.0 W cm^−2^) under 1064 nm laser irradiation for 10 min. ESR spectra of (I) DMPO/·OH, (J) DMPO/O_2_^·−^ and (K) TEMP/^1^O_2_ treated with physical mixture and CCS under different conditions. (L) Diagram of redox cycle reaction of GSH and ·OH conversion in CCS, (M) Photocatalytic mechanism of CCS heterostructure under 1064 nm laser irradiation. (N) The band structures of CCS, Cu_2_O, and Cu_2−_ *_x_* S and the energy positions of ·OH/H_2_O_2_ and O_2_^·−^/^1^O_2_ formation processes. (O) CLSM images of 4T1 cells stained with DCFH‐DA, LPO, H&E, terminal deoxynucleotidyl transferase dUTP nick end labeling (TUNEL), the corresponding GPX4 and HSP90 after various treatments. Scale bar: 50 µm. (P) Tumor growth curves in different treatment groups. (Q) Picture of separated tumor after treatment. Reproduced with permission [214]. Copyright 2024, Wiley‐VCH GmbH.

Gao et al. [214] developed a novel heterojunction of Cu_2_O/Cu_2−_ *_x_* S modified with gambogic acid and hyaluronic acid (CCS@GA@HA) for synergistic cancer treatment, combining PTT, PCT, CDT, and potential FT (Figure 12A). As illustrated in Figure 12B–E, the synthesized CCS exhibited high dispersion with an average diameter of 39.93 nm. The formation of the heterojunction was confirmed by the well‐distributed lattice fringes of Cu_2−_ *_x_* S (*d*_220_ = 0.151 nm) and Cu_2_O (*d*_220_ = 0.201 nm). UV–vis absorption and diffuse reflectance spectra indicated that the presence of Cu_2−_ *_x_* S provided CCS with excellent absorption capabilities in the NIR‐II region, enhancing tissue penetration and biosafety for phototherapy (Figure 12F,G). The photothermal performance of CCS was subsequently evaluated, demonstrating remarkable concentration‐ and time‐dependent properties within the measured range (Figure 12H). ESR spectroscopy was employed to investigate the mechanism of ROS generation by CCS heterostructures. As shown in Figure 12I, CCS combined with H_2_O_2_ exhibited a strong ·OH signal even in the absence of laser treatment, indicating its excellent Fenton‐like activity. Notably, this ·OH signal intensified under NIR irradiation. This enhancement could be attributed to two factors: (1) the increased temperature from the photothermal effects of CCS, which accelerated the Fenton‐like reaction; and (2) the favorable band structure of CCS, which facilitated ·OH production via photocatalysis. Additionally, strong characteristic peaks of O_2_^·−^ and ^1^O_2_ emerged in the CCS + H_2_O_2_ group only after NIR irradiation (Figure 12J,K), demonstrating the effective photocatalytic activity of CCS. The Fenton‐like activity of CCS consumed GSH and produced ·OH through the Cu^2+^/Cu^+^ redox cycle (Figure 12L). Moreover, the heterostructure enhanced photocatalytic activity under NIR irradiation, aided by matched energy levels and improved charge carrier separation efficiency (Figure 12M,N). No significant fluorescence enhancement was observed in the CCS‐ and CCS@GA@HA‐treated 4T1 cells compared to the control group. However, a notable increase in fluorescence intensity was detected upon NIR laser irradiation (Figure 12O). The strong ROS production and efficient GSH depletion likely triggered lipid peroxidation (LPO), as evidenced by the intense green fluorescence in the CCS@GA@HA + NIR group, which induced ferroptosis in tumor cells. Notably, GA significantly inhibited HSP90 activity, reducing tumor cell resistance to thermal ablation and enhancing the mild photothermal therapeutic effect of CCS. Hematoxylin and eosin (H&E) staining showed the most severe tumor cell damage in the CCS@GA@HA + NIR group, aligning with results depicted in Figure 12P,Q. Additionally, immunofluorescence staining revealed the lowest glutathione peroxidase 4 (GPX4) expression in the CCS@GA@HA + NIR group, which further confirmed ferroptosis in tumors. Thus, the simple fabrication of CCS@GA@HA enables a combination therapy of NIR‐triggered mild PTT, PCT, CDT, and FT, inspiring future designs of multifunctional nanoplatforms for synergistic tumor therapy.

**FIGURE 13:**
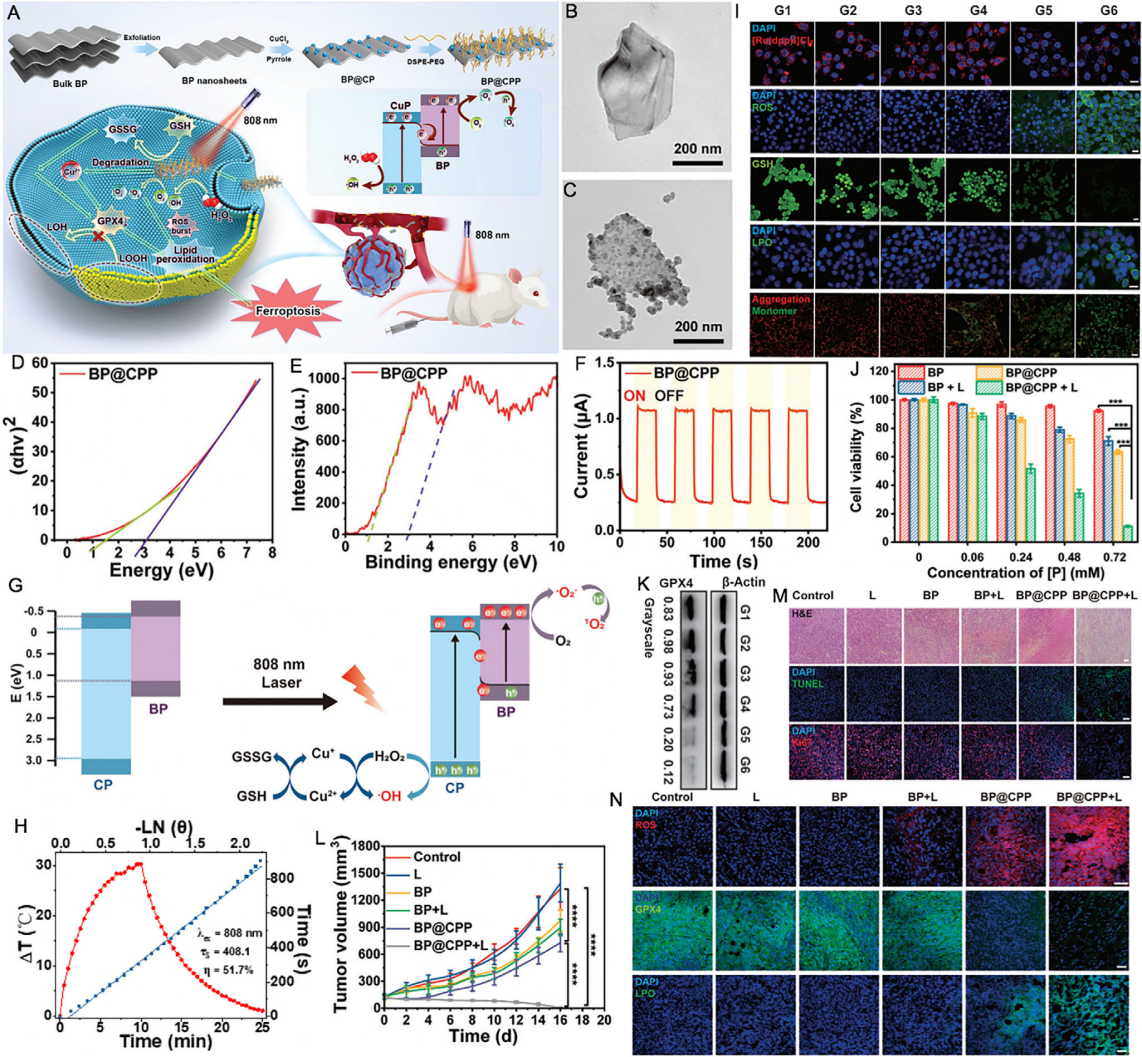
(A) The schematic illustration of synthesis procedures of BP@CPP for photo‐enhanced tumor ferroptosis therapy. TEM images of (B) BP and (C) BP@CP. (D) Bandgap of BP and CP estimated from Kubelka–Munk equation. (E) VB of BP@CPP calculated from XPS spectra. (F) Photocurrent curve of BP@CPP under 808 nm laser irradiation. (G) Schematic illustration of Z‐schemed heterojunction based on BP@CPP for ·O_2_^−^ and ^1^O_2_ generation, and GSH oxidation. (H) Photothermal heating and cooling curves of BP@CPP under 808 nm laser (1 W cm^−2^) and corresponding linear relationship between time and −ln (𝜃) from the cooling period. (I) CLSM images analysis of intracellular O_2_, ROS, GSH, and LPO levels in 4T1 cells under hypoxia condition after different treatments and CLSM images of mitochondrial membrane potential after different treatments. Scale bars: 20 µm. (J) Cell viability of 4T1 cells after different treatments with or without 808 nm laser irradiation (1.0 W cm^−2^, 5 min). (K) Western blot analysis of GPX4 protein in 4T1 cells after various treatments. (L) Tumor growth curves of 4T1 tumor‐bearing Balb/c mice subject to different treatments. (M) H&E, TUNEL, and Ki67 staining of tumor tissues in various groups. Scale bars: 50 µm. (N) In vivo ROS detection in the sections from tumor sand immunofluorescence staining of GPX4 protein and LPO detection in the sections from tumors. Scale bars: 50 µm. Data represent mean ± SD (*n* = 5). Reproduced with permission [215]. Copyright 2024, Wiley‐VCH GmbH.

Liu et al. [215] utilized the phosphates on the surface of black phosphorus (BP) to chelate Cu^2+^, subsequently creating a Cu^+^/Cu^2+^‐doped BP@polypyrrole heterojunction (denoted as BP@CP) through the in situ polymerization of pyrrole monomers (Figure 13A). As shown in Figure 13B,C, both BP and BP@CP exhibited similar sheet‐like morphology and dimensions, with lengths of ∼457.2 nm (BP) and ∼421.7 nm (BP@CP) and widths of ∼314.3 nm (BP) and ∼327.4 nm (BP@CP). However, BP@CP displayed a rougher surface due to the substantial deposition of CP. The enhanced catalytic performance of BP@CP was attributed to the Cu^2+^‐doped polypyrrole deposition, as confirmed by XPS analysis, solid‐state UV–vis–NIR diffuse reflectance spectroscopy, and photocurrent response measurements (Figure 13D–F). Upon reaching the tumor region, the PEGylated BP@CP (BP@CPP) facilitated a rapid reaction between the doped Cu^+^/Cu^2+^ ions and H_2_O_2_ present in the TME, resulting in the production of ·OH and O_2_. Cu^2+^ was able to react with GSH to generate glutathione disulfide (GSSG), leading to an increase in ROS concentration and a decrease in GSH levels within the tumor. More intriguingly, the Z‐scheme heterojunction nanostructure in BP@CPP facilitated effective separation of electron–hole pairs and prevented their recombination through charge migration at the interface under 808 nm laser irradiation. In this procedure, activated electrons in the VB of BP NSs catalyzed the conversion of generated O_2_ into O_2_^·−^, which were subsequently oxidized into ^1^O_2_. Concurrently, the activated holes in the CB of CP catalyzed the conversion of H_2_O_2_ into ·OH (Figure 13G). Additionally, BP@CPP efficiently converted light energy into heat for PTT, with a calculated PCE of 51.7%, enhancing the overall catalytic reactions (Figure 13H). With the continuous accumulation of ROS and the depletion of GSH, intracellular GPX4 became inactivated, leading to an elevation in LPO (Figure 13I–K). Such process enhanced charge migration and promoted ferroptosis in tumor cells, resulting in significant tumor suppression in vivo following a single drug injection and laser irradiation (Figure 13L–N). This study underscores that the synthesized BP@CPP heterojunction effectively integrates ROS bursts and GSH/GPX4 inactivation into a biocompatible nanoplatform, facilitating abundant LPO accumulation and thereby amplifying ferroptosis‐like cancer cell death.

##### 3.2.3.3 PTT/PCT/FT/IT

The advantages of combining PTT, PCT, FT, and IT in cancer treatment include enhanced tumor destruction through multiple mechanisms, which increases treatment efficacy. PTT and PCT provide immediate cell damage, while ferroptosis promotes sustained cell death, addressing tumor heterogeneity. This collaboration also facilitates the release of tumor antigens, boosting the immune response and improving the effectiveness of IT. Additionally, targeting different pathways can help circumvent resistance, offering a more comprehensive and potentially more effective cancer treatment strategy [216, 217, 218].

**FIGURE 14:**
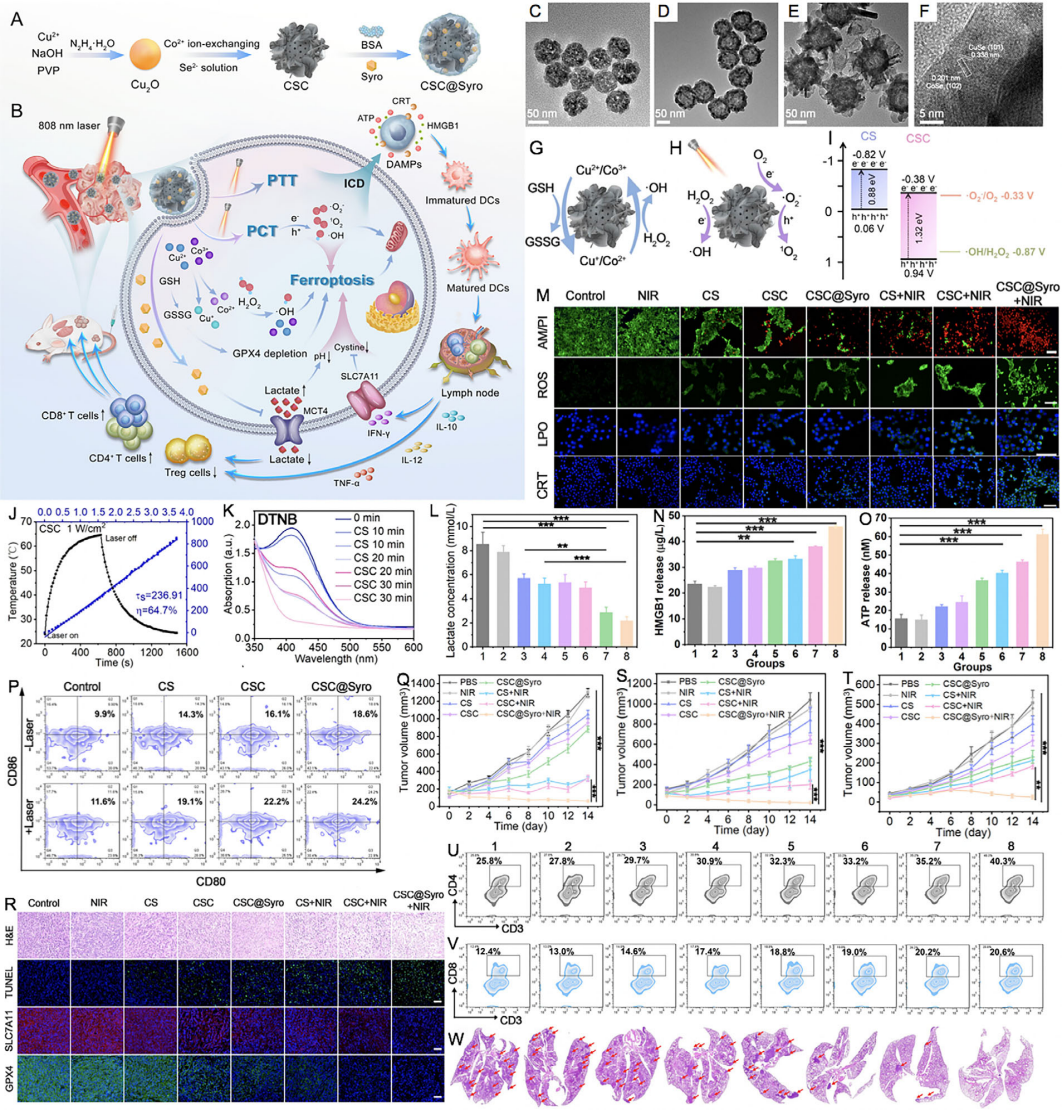
(A) Synthetic route of CSC@Syro and (B) the mechanism of potentiating antitumor IT induced by TME and NIR coactivatable synergistic PTT/PCT/FT and improved by LA metabolic reprogramming. TEM images of (C) Cu_2_O, (D) CS, and (E) CSC. (F) The HRTEM image of CSC. The graphic illustration of (G) the redox recycle reaction in CSC for converting GSH and ·OH, and (H) the photocatalytic mechanism of the CSC heterostructure under 808 nm laser irradiation. (I) The band structures of CS and CSC and the energy positions of the ·OH/H_2_O_2_ and O_2_^·−^/O_2_ generation process. (J) Temperature elevation and cooling curves of CSC solution (250 µg mL^−1^) after 10 min of irradiation (1.0 W cm^−2^) and linear regression data obtained from the cooling period. (K) Time‐dependent UV/vis absorption changes of DTNB treated with CS and CSC (100 µg mL^−1^) in PBS solution containing GSH (10 mM). (L) Quantitative analysis of extracellular LA levels after different treatments (1: control, 2: NIR, 3: CS, 4: CS + NIR, 5: CSC, 6: CSC + NIR, 7: CSC@Syro, 8: CSC@Syro + NIR). (M) Fluorescence images of 4T1 cells for calcein‐AM/PI, DCFH‐DA, LPO and CRT images in different groups. Scale bars: 50 µm. Statistical significance was calculated using Student′s *t* test: *p < 0.05, **p < 0.01, and ***p < 0.001. (N) ELISA measurement of HMGB1 released in the extracellular supernatant after different treatments (1: control, 2: NIR, 3: CS, 4: CSC, 5: CSC@Syro, 6: CS + NIR, 7: CSC + NIR, 8: CSC@Syro + NIR). (O) The release of ATP levels in the extracellular supernatant in different groups. (P) Flow cytometry analysis for CD80 and CD86 expression on DC cells in the superstratum. Statistical significance was calculated using Student′s *t* test: *p < 0.05, **p < 0.01, and ***p < 0.001. (Q) Time‐dependent variations of tumor volumes after mice treated under various conditions. (R) Representative immunofluorescence images of H&E, TUNEL, SLC7A11, and GPX4 tumor dissection obtained from excised tumors with different treatments (scale bar: 50 µm). Statistical significance was calculated using Student′s *t* test: *p < 0.05, **p < 0.01, and ***p < 0.001. (S, T) The primary and distant tumor sizes after various treatments. The flow cytometry of (U) CD3^+^CD4^+^ T cells and (V) CD3^+^CD8^+^ T cells in distant tumors after various treatments for 14 days. (W) H&E staining of representative lung sections in antimetastatic studies. Statistical significance: *p < 0.05, **p < 0.01, and ***p < 0.001. Reproduced with permission [219]. Copyright 2023, American Chemical Society.

Yang et al. [219] developed a hollow mesoporous CuSe/CoSe_2_@syrosingopine (CSC@Syro) heterostructure as a versatile nanoadjuvant for multi‐modal cancer therapy (Figure 14A,B). Monodisperse hollow CuSe (CS) was synthesized using Cu_2_O as a sacrificial precursor based on the Kirkendall effect. CSC heterostructures were then formed by partially exchanging Co^2+^ with the Cu_2_O precursor, followed by reaction with Se^2−^ (Figure 14C–E). HRTEM revealed CuSe (*d*_101_ = 0.338 nm) and CoSe_2_ (*d*_102_ = 0.201 nm) lattice fringes, confirming heterojunction formation (Figure 14F). As shown in Figure 14G–I, CSC produced ·OH via Fenton‐like reactions and, under 808 nm light, generated O_2_^·−^ and ^1^O_2_ from photogenerated electrons. The introduction of H_2_O_2_ and NIR laser enhanced ·OH signals through reactions with photoelectrons. The larger specific surface area of CSC provided abundant active sites, enhancing laser absorption and utilization. Temperature monitoring showed a 38.4°C increase for CSC (250 µg mL^−1^), with a PCE of 64.7% (Figure 14J). The presence of high‐valent Cu^2+^ and Co^3+^, along with Se‐Se bonds, allowed CSC nanoparticles to consume GSH, as detected using the (5,5‐dithio‐bis‐(2‐nitrobenzoic acid) (DTNB) probe (Figure 14K). The pHrodo Green AM indicator confirmed that the CSC@Syro nanoplatform effectively regulated LA metabolism and remodeled the TME (Figure 14L). The live/dead cell staining assay demonstrated significant cell lethality, particularly in the CSC@Syro + NIR group (Figure 14M). Additionally, LPO resulting from GPX4 inactivation was detected using the Liperfluo probe, with the brightest green fluorescence in the CSC@Syro + NIR group. The CSC@Syro nanoplatform could induce ICD in 4T1 cells, stimulating immune defense to inhibit cancer recurrence and metastasis. As shown in Figure 14N, ELISA revealed a sharp increase in high mobility group box 1 protein (HMGB1) release following CSC@Syro + NIR treatment. Additionally, enhanced calreticulin (CRT) exposured on 4T1 cells, indicated by increased green fluorescence in the cytoplasm was observed (Figure 14M). ATP levels, serving as a “find me” signal, quadrupled in the CSC@Syro + NIR group, promoting specific antitumor immune responses (Figure 14O). Flow cytometry results demonstrated a 24.2% increase in CD80/CD86 costimulatory molecule expression, confirming enhanced DC maturation due to DAMP release (Figure 14P). Notably, tumors were nearly ablated in the CSC@Syro + NIR group, highlighting the effectiveness of the synergistic PTT/PCT/FT/IT approach (Figure 14Q). H&E and TUNEL assays showed significant tumor cell shrinking and increased green fluorescence in the CSC@Syro + NIR group, indicating strong apoptosis induction (Figure 14R). The reduced fluorescence confirmed the inhibition of SLC7A11, which disrupted cystine/glutamate transport, decreased GSH production, and triggerred LPO to promote ferroptosis. Additionally, decreased GPX4 expression further indicated ferroptosis occurrence. More importantly, the CSC@Syro + NIR group significantly suppressed both primary and distant tumors, demonstrating strong immune potential against metastasis (Figure 14S,T). Flow cytometry revealed notable CD4^+^/CD8^+^ T cell infiltration in distant tumors to aid the elimination of metastatic cells, which were consistent with the lung metastasis inhibition trend from H&E staining (Figure 14U–W). Thus, the CSC@Syro platform effectively inhibited primary tumor growth and delayed metastasis, offering a promising strategy for enhanced IT through tumor metabolic regulation.

##### 3.2.3.4 PTT/PCT/GT/IT

**FIGURE 15:**
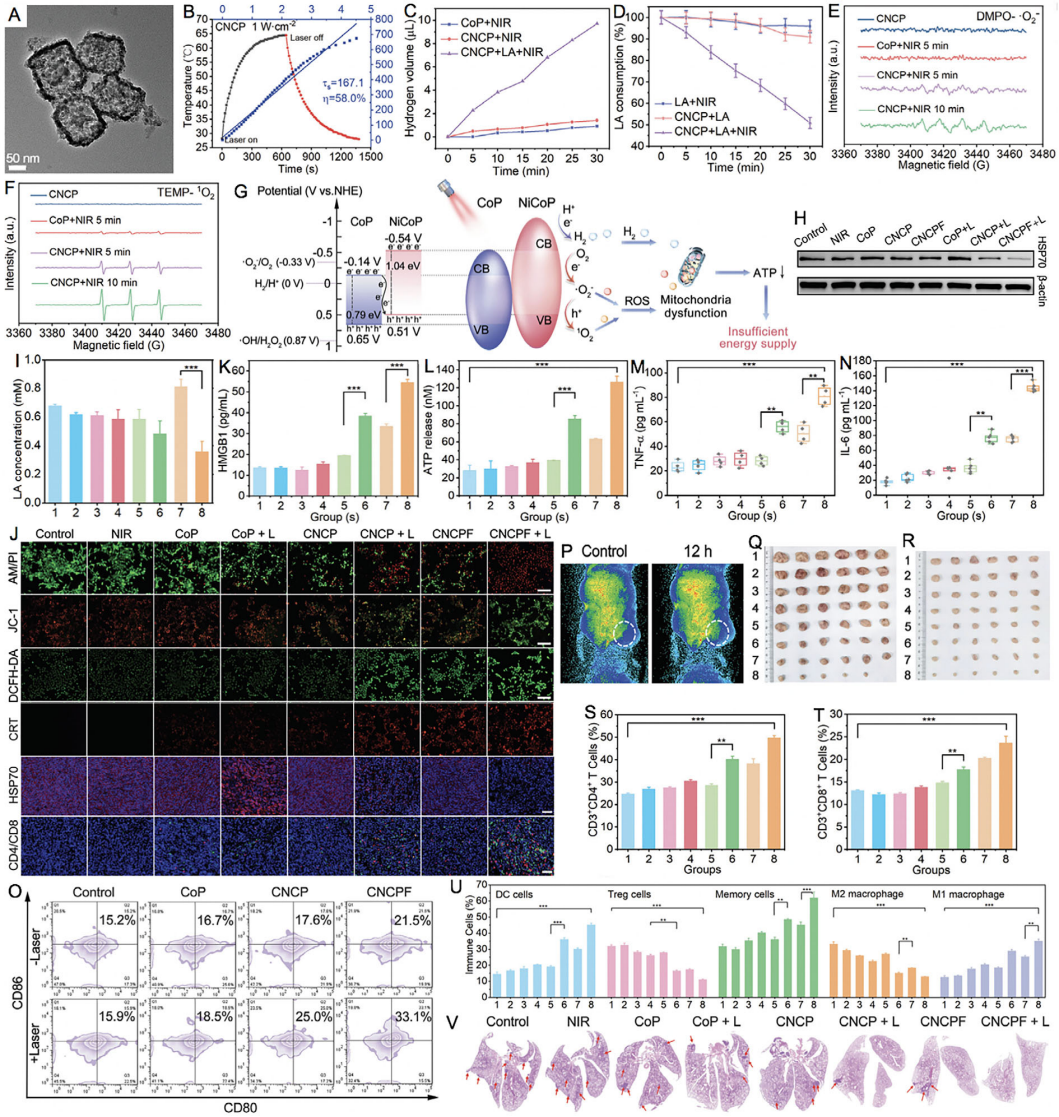
(A) TEM image of CNCP NPs. (B) Temperature change of CNCP solution (200 µg mL^−1^) irradiated with 808 nm laser (1.0 W cm^−2^) for 10 min, followed by ambient cooling to obtain linear regression data for calculating the PCE. (C) Photocatalytic H_2_ production detected by gas chromatography under different conditions. (D) LA consumption after different treatments evaluated by LA content assay (*n* = 3). Data are presented as mean ± SD. ESR spectra of DMPO/·O_2_^−^ (E) and TEMP/^1^O_2_ (F) treated with CoP and CNCP under 808 nm laser irradiation. (G) Band structures of CNCP heterojunction and the energy positions of ·O_2_^−^/O_2_, H_2_/H^+^, and ·OH/H_2_O_2_ generation process, and the schematic therapeutic pathway of H_2_ and ROS effects on mitochondria. (H) Western blots of HSP70 in 4T1 cells with different treatments. (I) Quantitative analysis of extracellular and intracellular LA levels after different treatments (1: control, 2: NIR, 3: CoP, 4: CoP + L, 5: CNCP, 6: CNCP + L, 7: CNCPF, 8: CNCPF + L). Data are presented as mean ± SD (*n* = 3). Statistical significance was calculated using Student's *t*‐test: *p < 0.05, **p < 0.01, and ***p < 0.001. (J) Representative immune‐fluorescence images of calcein‐AM/PI, JC‐1, DCFH‐DA, and CRT in 4T1 cells after different treatments; HSP70 and CD4^+^/CD8^+^ T cells in tumor dissection obtained from excised tumors with different treatments (Scale bar = 50 µm). (K) The extracellular release of HMGB1 in the supernatant measured by ELISA assay after treated with various groups (1: control, 2: NIR, 3: CoP, 4: CoP + L, 5: CNCP, 6: CNCP + L, 7: CNCPF, 8: CNCPF + L). (L) The levels of ATP release of 4T1 cells after different treatments. ELISA assay measurement of TNF‐𝛼 (M) and IL‐6 (N) released in the medium during the transwell experiment. (O) Flow cytometric analysis of CD80/CD86 expression to assess DC maturation. (P) In vivo MR images of 4T1 tumor‐bearing mice after intravenously injected with PBS or CNCP NPs for 12 h. (Q, R) Photograph of dissected primary tumors and distant tumors after various treatments for 14 days (1: control, 2: NIR, 3: CoP, 4: CoP + L, 5: CNCP, 6: CNCP + L, 7: CNCPF, 8: CNCPF + L). The semiquantitative analysis from flow cytometry detection of CD3^+^CD4^+^ T cells (S) and CD3^+^CD8^+^ T cells (T). (U) Statistic histogram from flow cytometry analysis of CD11c^+^CD80^+^CD86^+^ DCs in the lymph node, CD3^+^CD4^+^Foxp3^+^ Treg cells in spleens, CD8^+^CD44^+^CD62L^+^ central memory T cells in spleens, and M1/M2 macrophages in tumors. (V) H&E staining of representative lung sections in antimetastatic studies. Reproduced with permission [220]. Copyright 2024, Wiley‐VCH GmbH.

Yang et al. [220] developed a NIR light‐activated immunoadjuvant based on cobalt phosphide/nickel‐cobalt phosphide (CoP/NiCoP, denoted as CNCP) nanophotocatalysts, loaded with fluvastatin sodium (Flu), a monocarboxylate transporter 4 (MCT4) inhibitor, to enhance photocatalytic H_2_ therapy and photoimmunotherapy. TEM revealed a unique yolk–shell structure, which improved structural stability and facilitated reactant transport across interfaces (Figure 15A). Under 808 nm light irradiation, CNCP exhibited strong photothermal effects and efficient photocatalytic activity. With a PCE of 58.0%, CNCP outperformed CoP (43.1%) due to its yolk–shell architecture, which enhanced light absorption through internal reflection and increased active site density (Figure 15B). The energy band structure of CNCP was well‐suited for H_2_ and ROS generation. Upon laser irradiation, photoexcited electrons produced O_2_^·−^, which were subsequently converted to ^1^O_2_ via photogenerated holes. A significant decrease in LA levels was observed in the CNCP solution after NIR exposure, confirming the role of LA as an effective sacrificial agent in photocatalytic reactions (Figure 15C–G). The photothermal effect promoted targeted Flu release, effectively inhibiting MCT4‐mediated LA efflux, a hallmark of glycolytic cancer cells. Intracellular LA served as a sacrificial donor in photocatalytic H_2_ production, enhancing efficiency while depleting LA within tumor cells. The synergistic action of ROS and H_2_ disrupted mitochondrial function, impairing ATP synthesis required for HSP production, thereby enabling mild PTT. In vitro, phototoxicity correlated with ROS generation and mitochondrial membrane potential changes. Nearly all CNCPF + L‐treated cells exhibited red fluorescence with no green signal, indicating maximal therapeutic efficacy (Figure 15H–J). This combinatorial approach induced robust ICD, as evidenced by elevated CRT exposure, reduced HMGB1, and increased ATP release (Figure 15J–L). CNCPF + L treatment also significantly boosted mature DC populations (33.1%), surpassing other groups (Figure 15M–O). Elevated TNF‐α and IL‐6 levels further confirmed strong immune activation. Additionally, LA depletion in the TME alleviated immunosuppression, enhancing IT efficacy. In vitro, excised tumor images demonstrated near‐complete eradication with CNCPF + L (Figure 15Q,R). Notably, CNCPF + L treatment markedly reduced HSP70 immunofluorescence intensity, attributed to enhanced H_2_ and ROS production during PCT. This potent immune response also promoted DC maturation and T‐cell infiltration, suppressing tumor growth and lung metastasis (Figure 15J,S–V). Furthermore, owing to Co^2+^ release, CNCPF showed promise as a T_1_‐weighted magnetic resonance imaging (MRI) contrast agent, with increased relative signal intensity (RSI) at the tumor site 12 h post‐injection. This approach offers a promising new paradigm for developing highly precise and safe immunotherapeutic treatments.

## 4 Conclusions and Perspectives

Photocatalysis has emerged as a transformative approach in cancer theranostics over the past decade. To enhance therapeutic efficacy and address existing limitations, research has increasingly focused on developing integrated multi‐energy photocatalytic systems, particularly piezo‐photocatalysis and photothermal‐photocatalysis. These advanced systems offer distinct yet complementary advantages: Piezo‐photocatalysis synergizes piezoelectric effects with photocatalysis, demonstrating superior efficiency, precise targeting, and enhanced safety compared to conventional methods. By harnessing both mechanical and optical stimuli, this approach enables effective treatment of deep‐seated (e.g. pancreatic, liver) and hypoxic tumors through enhanced ROS generation and improved tissue penetration. Conversely, photothermal‐photocatalysis integrates light‐induced hyperthermia with photocatalytic reactions, accelerating reaction kinetics and amplifying ROS production. Particularly effective for superficial tumors (e.g. melanoma) and immunologically cold microenvironments, this approach combines localized heating with ICD for enhanced therapeutic outcomes. While each system presents unique strengths, they also face specific limitations: piezo‐photocatalysts are constrained by material availability and dependence on external mechanical energy, whereas photothermal systems encounter challenges with deep‐tissue penetration and potential thermal resistance. A comparative analysis highlights their tumor‐specific applicability, underscoring the importance of developing adaptive, multi‐energy designs tailored to tumor characteristics including depth, microenvironment, and metastatic potential (Table 2).

**TABLE 2:** A comparison of multi‐energy photocatalysis for different tumor models.

Feature	Piezo‐photocatalysis	Photothermal‐photocatalysis
Primary stimuli	Mechanical (US, pressure) + Light	Light (NIR)
Key advantages	Deep tissue penetration (suitable for deep‐seated tumors); enhanced ROS generation under mechanical stress; reduced off‐target effects	Localized hyperthermia enhances ROS production; improves drug release in chemotherapy‐PTT; immune activation via PTT‐induced ICD
Tumor models	Solid tumors (e.g. breast, liver); hypoxic tumors (benefits from mechanical‐enhanced ROS); ultrasound‐accessible regions (e.g. pancreatic tumors)	Superficial or NIR light‐accessible tumors (e.g. melanoma, skin cancer); tumors with high thermal sensitivity (e.g. glioblastoma); metastatic tumors (combined with IT)
Limitations	Dependent on external mechanical source; limited in non‐piezoelectric materials	Limited penetration depth in deep tumors; potential thermal resistance in some cancers

This work provides an overview of recent developments in multi‐energy integrated photocatalysis for cancer therapy. It begins with an introduction to the principles of piezo‐photocatalysis and piezo‐photocatalysts in the field of cancer treatment, including single‐modal piezoelectric‐enhanced PDT, dual‐modal SPDT, and triple‐modal HT/GT/chemotherapy. The discussion then shifts to photothermal‐photocatalysis, focusing on the combination of different treatment approaches to form dual‐modal systems, such as PTT/PCT and PTT/PDT, as well as more complex combinations like triple‐modal PTT/PCT/chemotherapy, PTT/PCT/CDT, PTT/PCT/GT, and PTT/PCT/IT. Further, it explores tetral‐modal therapies, including PTT/PCT/CDT/chemotherapy, PTT/PCT/CDT/FT, PTT/PCT/FT/IT, and PTT/PCT/GT/IT (Table 3). Despite notable progress in the field of multi‐energy integrated photocatalysis, several critical challenges remain that must be overcome before these approaches can be translated into clinical practice.

**TABLE 3:** Summary of different piezo‐photocatalysts and photothermal‐photocatalysts for cancer treatment.

Material	Tumor model	Treatment modality	Treatment parameter	Reference
BGO	143B tumor	Piezo‐enhanced PDT	NIR: 808 nm, 1 W cm^−2^, 5 min; US: 1 MHz, 2 W cm^−2^, 5 min	[112]
Au@PEG‐ZnO	LLC‐luc tumor	Piezo‐enhanced PDT	UV: 3 mW, 7 min; US: 500 KHz, 2 W, 7 min	[113]
ZnSnO_3_@UCNPs	4T1 tumor	SPDT	NIR: 980 nm, 0.4 W cm^−2^, 2.5 min; US: 1 MHz, 0.96 W cm^−2^, 2.5 min	[125]
CPL@M	HeLa tumor	HT/GT/chemotherapy	Luminol‐excited chemiluminescence; US: 3 W, 60 s	[135]
TiSe_2_	HeLa tumor	PTT/PCT	808 nm, 1 W cm^−2^, 10 min	[156]
BiO*_x_* Cl@PEG	4T1 tumor	PTT/PDT	1064 nm, 1 W cm^−2^, 5 min	[160]
ox‐CDs	4T1 tumor	PTT/PCT	730 nm, 1 W cm^−2^, 5 min	[165]
BS/Au@PP‐DOX	4T1 tumor	PTT/PCT/chemotherapy	808 nm, 1 W cm^−2^, 10 min	[175]
RGD‐dpGCS	U87‐MG tumor	PTT/PCT/CDT	1064 nm, 1 W cm^−2^, 5 min	[184]
ACP	4T1 tumor	PTT/PCT/CDT	1064 nm, 0.75 W cm^−2^, 10 min	[185]
ENBS‐PEG@L‐TiO_2_‐GNR	4T1 tumor	PTT/PDT/GT	808 nm, 0.8 W cm^−2^, 10 min	[195]
BZP	4T1 tumor	PTT/PCT/IT	730 nm, 1 W cm^−2^, 8 min	[209]
CuSe/NC‐DOX‐DNA	HeLa tumor	PTT/PCT/CDT/chemotherapy	808 nm, 1 W cm^−2^, 10 min	[213]
CCS@GA@HA	4T1 tumor	PTT/PCT/CDT/FT	1064 nm, 1 W cm^−2^, 10 min	[218]
BP@CPP	4T1 tumor	PTT/PCT/CDT/FT	808 nm, 1 W cm^−2^, 10 min	[219]
CSC@Syro	4T1 tumor	PTT/PCT/FT/IT	808 nm, 1 W cm^−2^, 5 min	[223]
CNCPF	4T1 tumor	PTT/PCT/GT/IT	808 nm, 0.5 W cm^−2^, 5 min	[224]

In piezo‐photocatalysis, performance is enhanced by combining piezoelectric materials with semiconductor photocatalysts, each contributing its distinct piezoelectric properties and photocatalytic activity. Typically, high‐frequency, high‐power ultrasonic vibrations are used to induce mechanical excitation in these systems. However, such vibrations can compromise the bond between the two materials, leading to a decline in both cyclic stability and overall efficiency. To mitigate this issue, integrating semiconductor photocatalysis and piezoelectric polarization within a single material may help eliminate the stability challenges observed in composite structures. Additionally, the development of piezocatalysts responsive to low‐frequency mechanical energy is crucial for optimizing catalytic performance. While improvements in catalytic activity have been linked to the electric fields generated within the system, which aid in charge carrier separation, the full potential of the synergistic interactions between piezoelectric and semiconductor properties remains inadequately explored. Several factors, including modifications in band structure, piezoelectric coefficients, dielectric constants, conductivity, and interfacial charge transfer dynamics, have yet to be thoroughly investigated. Consequently, further experimental studies and theoretical models, employing advanced characterization methods, are necessary to advance the design of more efficient piezophotocatalytic systems.

Achieving an optimal balance between heat generation and ROS production in photothermal‐photocatalysis nanosystems is essential for maximizing therapeutic efficacy while minimizing side effects in cancer treatment. For example, materials with lower *E*_g_ values, such as N‐ or C‐doped TiO_2_, promote enhanced electron–hole pair separation, leading to increased ROS generation [221, 222]. In contrast, materials with higher *E*_g_ values, like molybdenum disulfide (MoS_2_), are more effective at generating heat due to their superior light absorption in the NIR region [223]. Additionally, smaller nanoparticles have higher surface area‐to‐volume ratios, which facilitates better interaction with oxygen molecules and enhances catalytic efficiency for ROS production. However, these smaller particles tend to generate less heat compared to larger nanoparticles [224]. Therefore, nanoparticles with appropriate *E*_g_ and intermediate sizes (10–50 nm) are expected to be an ideal compromise, enabling efficient light absorption and catalytic activity for ROS generation, while also producing sufficient heat for effective photothermal treatment.

Both PTT and PCT face significant challenges due to the limited penetration depth of light. Although NIR light, a common choice in phototherapy, penetrates tissues more effectively than visible light, its ability to reach deeper tissues, especially in dense or large tumors, remains constrained. This limitation undermines the effectiveness of photothermal‐photocatalytic treatments for larger or more deeply located cancers. To address this, the development of novel NIR‐responsive photocatalysts via strategies such as band‐gap engineering, heterostructural design, and co‐catalyst optimization is essential [225, 226]. Equally important is improving the efficiency of laser delivery. Recent innovations in optical fibers and implantable light delivery systems have made it possible to direct NIR‐II light precisely to deeper tumor regions, thereby enhancing the localized treatment outcome [227, 228].

The TME is marked by distinctive features, such as acidic pH and low oxygen levels, both of which can significantly impact heat generation and ROS production. Effectively managing these factors is crucial to enhancing the therapeutic efficacy of treatments. Semiconductor nanoparticles can be specifically designed to respond to the acidic conditions (pH 6.5–7.0), promoting ROS generation under such conditions while maintaining their photothermal capabilities. However, hypoxia, a common characteristic of tumors, can impair the efficiency of photocatalytic ROS production. To overcome this challenge, photocatalysts can be incorporated with nanoparticles capable of oxygen release, such as perfluorocarbons [229] or oxygen‐generating materials [230], to alleviate oxygen deficiency, thereby enhancing ROS production without necessitating excessive light exposure or heat generation. It is noteworthy that real‐time monitoring of both temperature and ROS levels within the tumor enables precise treatment adjustments. Advanced imaging techniques, including thermal imaging for temperature measurement and FLI for ROS detection, allow clinicians to assess the dynamic balance between heat and ROS generation during therapy [231, 232]. This continuous feedback enables the fine‐tuning of treatment parameters, such as light intensity, exposure time, and wavelength, to optimize the combined photothermal and photocatalytic effects for maximal therapeutic benefit.

The application of photocatalytic materials in medicine necessitates careful consideration of their biosafety profiles. In the case of piezo‐photocatalysts, current research primarily revolves around piezotronic materials like BaTiO_3_ and lead zirconate titanate (PZT). However, these materials may present biocompatibility challenges in clinical settings. To address this, future investigations should explore the development of alternative piezotronic materials, such as lead‐free variants, composite materials, and organic piezotronic substances. Furthermore, when nanomaterials are administered into the bloodstream, they often encounter rapid clearance by the immune system, limiting their ability to reach tumor sites. Understanding the biodistribution and pharmacokinetics of these multi‐energy integrated photocatalysts is essential to optimize their accumulation at tumor sites while preventing excessive buildup in non‐target tissues or organs. One effective approach to improving targeting and therapeutic outcomes is surface functionalization. Coating nanoparticles with PEG or tumor‐specific units can not only boost their biocompatibility and extend circulation time but also improve their selective accumulation in tumors, thereby maximizing the therapeutic benefits while minimizing potential side effects [233, 234]. Future investigations should prioritize assessing the long‐term toxicity of multi‐energy integrated photocatalysts within biological systems many inorganic nanosystems suffer from low biodegradability.

Tumors exhibit considerable heterogeneity, which encompasses differences in cellular composition, genetic mutations, and the TME. This variability can lead to inconsistent responses to multi‐energy integrated photocatalysts, both within different regions of a single tumor and between distinct tumors. Some tumor areas may display higher resistance to treatment, thereby limiting overall therapeutic efficacy. Although triple‐modal and tetra‐modal approaches offer the potential for synergistic effects, they also introduce complexities related to treatment optimization and the risk of negative interactions between the various modalities. Achieving an optimal balance and carefully fine‐tuning the parameters for each modality is essential to maximize therapeutic effectiveness while minimizing adverse effects. A key objective for multi‐energy integrated photocatalysts is their eventual industrial‐scale application. To realize this goal, future research efforts should emphasize feasibility studies, cost‐effectiveness analyses, and the development of scalable manufacturing processes to support large‐scale production.

## Author Contributions

**Li Zhang**: conceptualization, investigation, resources, writing‐original draft preparation, writing‐review and editing, visualization, funding acquisition. **Jun Liu**: investigation, writing‐original draft preparation, visualization. **Yuxuan Xiong**: resources, writing‐review and editing, visualization. **Guangfu Liao**: conceptualization, investigation, resources, writing‐original draft preparation, writing‐review and editing, supervision. **Yonggang Lv**: conceptualization, investigation, resources, writing‐original draft preparation, writing‐review and editing, supervision, funding acquisition. All authors have read and agreed to the published version of the manuscript.

## Conflicts of Interest

The authors declare no conflicts of interest.

## Data Availability

The authors have nothing to report.
